# Supplementary figures and images for: Biological insights from multi-omic analysis of 31 genomic risk loci for adult hearing difficulty
Source: PLoS Genet. 2020 Sep 28;16(9):e1009025. doi: 10.1371/journal.pgen.1009025 (PMC7544108; doi:10.1371/journal.pgen.1009025)

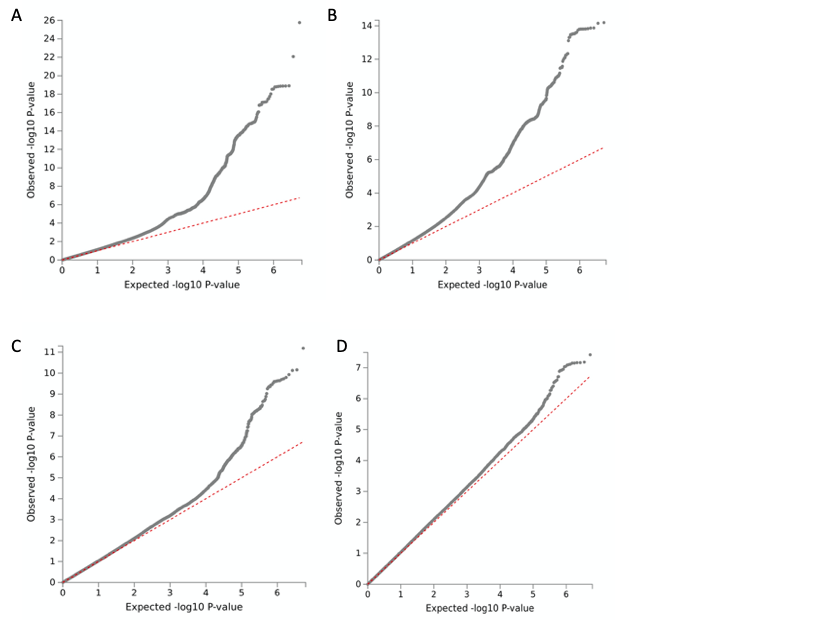

Supplement: S1 Fig — X-axis indicates the expected distribution of p-values for the associations of SNPs with each treat in the absence of true associations or confounding effects. y-axis indicates the observed distribution of p-values. A. 2247_1: “Hearing difficulty/problems: yes” B. 2257: “Hearing difficulty/problems with background noise” C. 3393: “Hearing aid user” D. 4803_11: “Tinnitus: Yes, now most or all of the time.” (TIF) [file pgen.1009025.s001.tif]

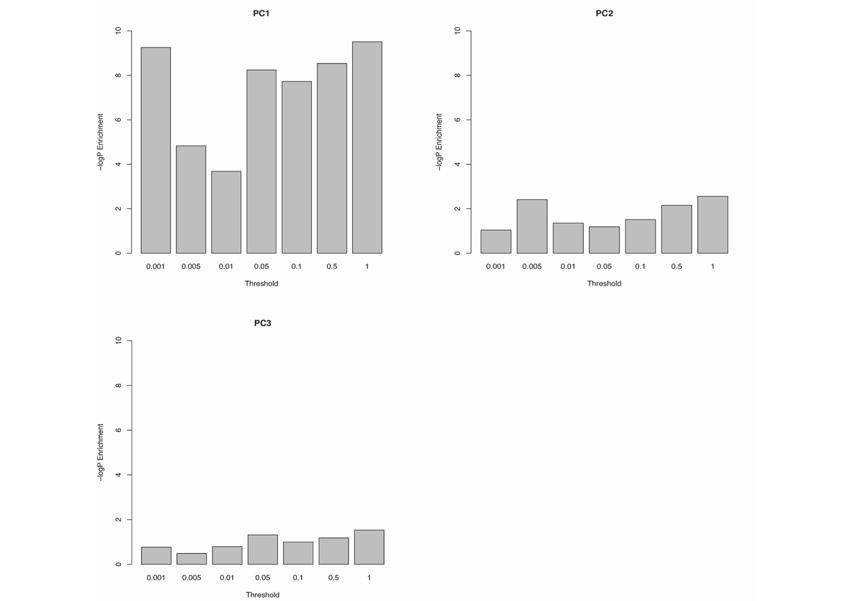

Supplement: S3 Fig — Polygenic risk scores (PRS) were calculated with PRSice-2[23], defined as the weighted sum of risk-associated SNPs from the UK Biobank hearing difficulty MTAG summary statistics and using the p-value cutoffs indicated on the x-axis. Y-axis indicates the -log10(p-value) from a test of whether each PRS score predicts binaural hearing thresholds in an independent sample of 1,472 Belgian adults[22]. Binaural hearing thresholds across a range of frequencies were summarized by principal component analysis, with principal component 1 (PC1) corresponding to the overall hearing capacity, PC2 corresponding to whether the audiogram is flat or sloping from low to high frequencies, and PC3 providing a measure of its convexity. (TIF) [file pgen.1009025.s003.tif]

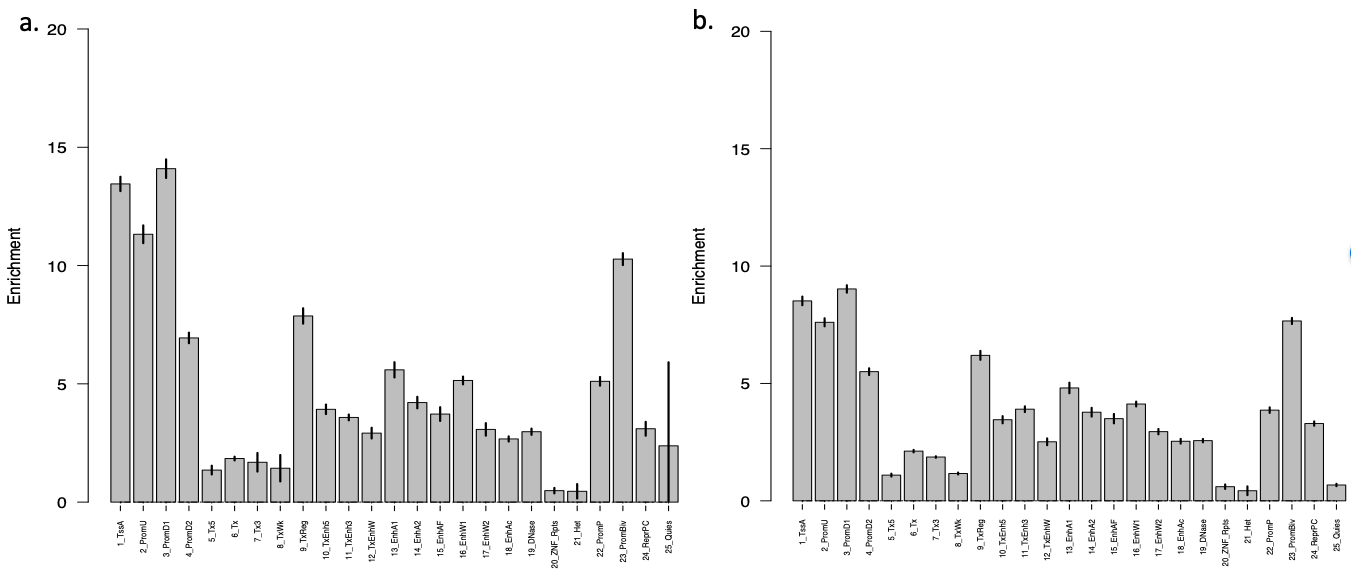

Supplement: S4 Fig — We predicted genomic regions that may be involved in gene regulation in the human cochlea based on homology to regions of open chromatin that we identified in epithelial (a) and non-epithelial cells (b) from mouse cochlea. To evaluate whether these human genomic regions correspond to true gene regulatory regions, we tested for overlap with chromatin states in 111 human tissues and cell types from the ROADMAP Epigenome Mapping Consortium. Y-axis indicates the fold enrichment (mean +/- standard error) within each chromatin state from a 25-state ChromHMM model: 1_TssA = Active TSS; 2_PromU = Promoter Upstream TSS; 3_PromD1 = Promoter Downstream TSS 1; 4_PromD2 = Promoter Downstream TSS 2; 5_Tx5 = Transcribed -5’ preferential; 6_Tx = Strong transcription; 7_Tx3 = Transcribed– 3’ preferential; 8_TxWk = Weak transcription; 9_TxReg = Transcribed and regulatory (Prom/Enh); 10_TxEnh5 = Transcribed 5’ preferential and Enh; 11_TxEnh3 = Transcribed 3’ preferential and Enh; 12_TxEnhW = Transcribed and Weak Enhancer; 13_EnhA1 = Active Enhancer 1; 14_EnhA2 = Active Enhancer 2; 15_EnhAF = Active Enhancer Flank; 16_EnhW1 = Weak Enhancer 1; 17_EnhW2 = Weak Enhancer 2; 18_EnhAc = Primary H3K27ac possible Enhancer; 19_DNase = Primary DNase; 20_ZNF_Rpts = ZNF genes & repeats; 21_Het = Heterochromatin; 22_PromP = Poised Promoter; 23_PromBiv = Bivalent Promoter; 24_ReprPc = Repressed Polycomb; 25_Quies = Quiescent/Low. (TIF) [file pgen.1009025.s004.tif]

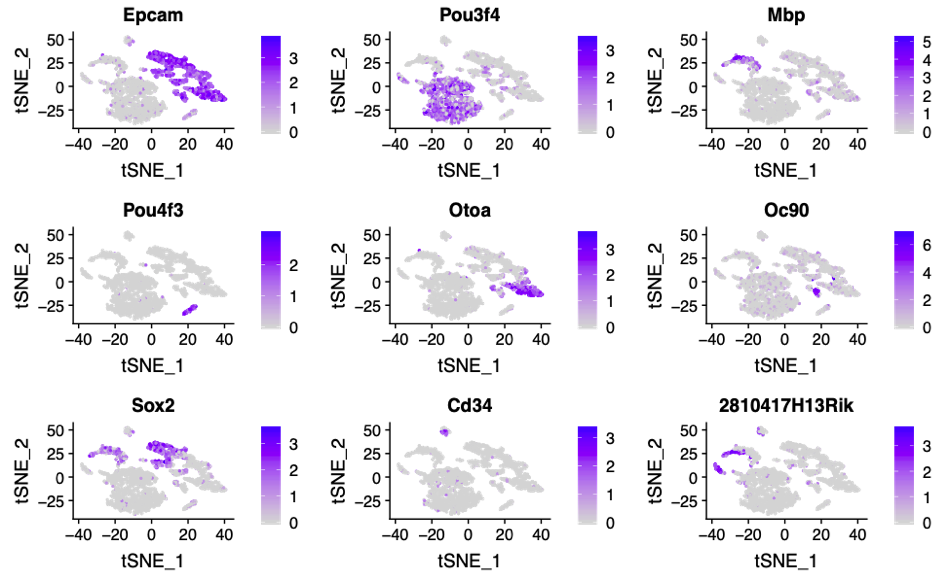

Supplement: S5 Fig — Expression patterns of canonical marker genes used to assign cell type labels to clusters of transcriptionally similar cells in single-cell RNA-seq of postnatal day 2 mouse cochlea. X- and y-axes indicate the positions of cells in a reduced dimensional space defined by t-stochastic neighbor embedding (tSNE), with all plots here and in Fig 5 displaying the cells using the same tSNE coordinates. Canonical marker gene specificities: Epcam, epithelial cells; Pou3f4, mesenchymal cells; Mbp, glia (oligodendrocytes); Pou4f3, sensory hair cells; Otoa, medial interdental cells; Oc90+ cells; Sox2, sensory epithelium supporting cells; Cd34, vascular cells; and 2810417H13Rik+, a marker of mesenchymal cells undergoing cell division. (TIF) [file pgen.1009025.s005.tif]

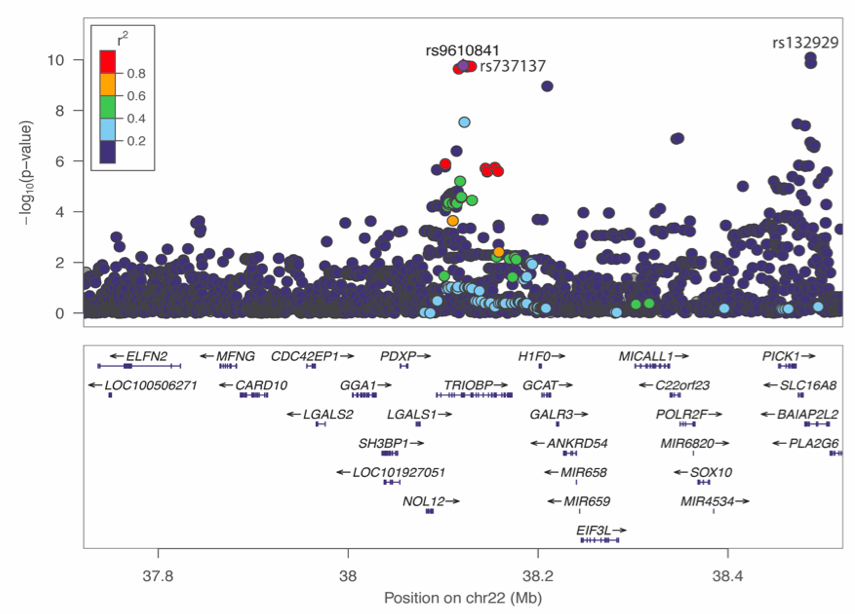

Supplement: S6 Fig — Independently significant SNPs at the chr22q13.1 risk locus (rs739137 and rs132929) were in strong LD (r2 > 0.9) with three protein-coding variants in the genes TRIOBP and BAIAP2L2. rs9610841 (TRIOBP Asn863Lys) and rs5756795 (TRIOBP Phe1187Leu) were in strong LD with each other, whereas rs17856487 (BAIAP2L2 Cys252Arg) was not in LD with any SNP predicted to impact TRIOBP. (TIF) [file pgen.1009025.s006.tif]
