# Supplementary material for: Biological insights from multi-omic analysis of 31 genomic risk loci for adult hearing difficulty
Source: PLoS Genet. 2020 Sep 28;16(9):e1009025. doi: 10.1371/journal.pgen.1009025 (PMC7544108; doi:10.1371/journal.pgen.1009025)

Locus Zoom Plots of Hearing Difficulty Loci

rsID: rs7525101

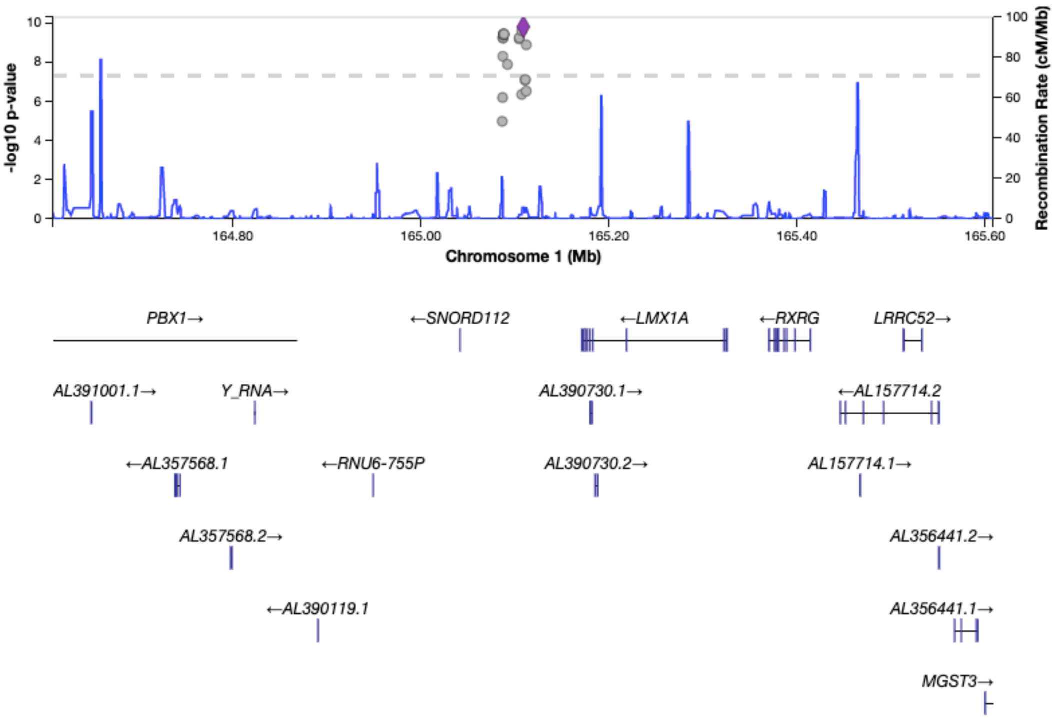

rsID: rs2941580

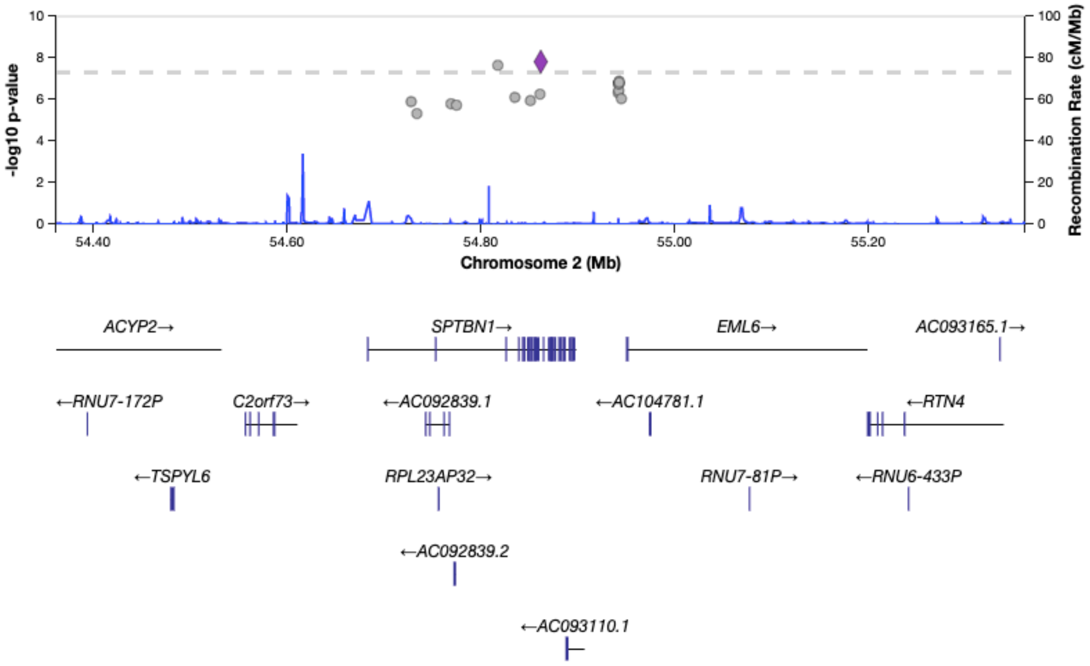

rsID: rs741475

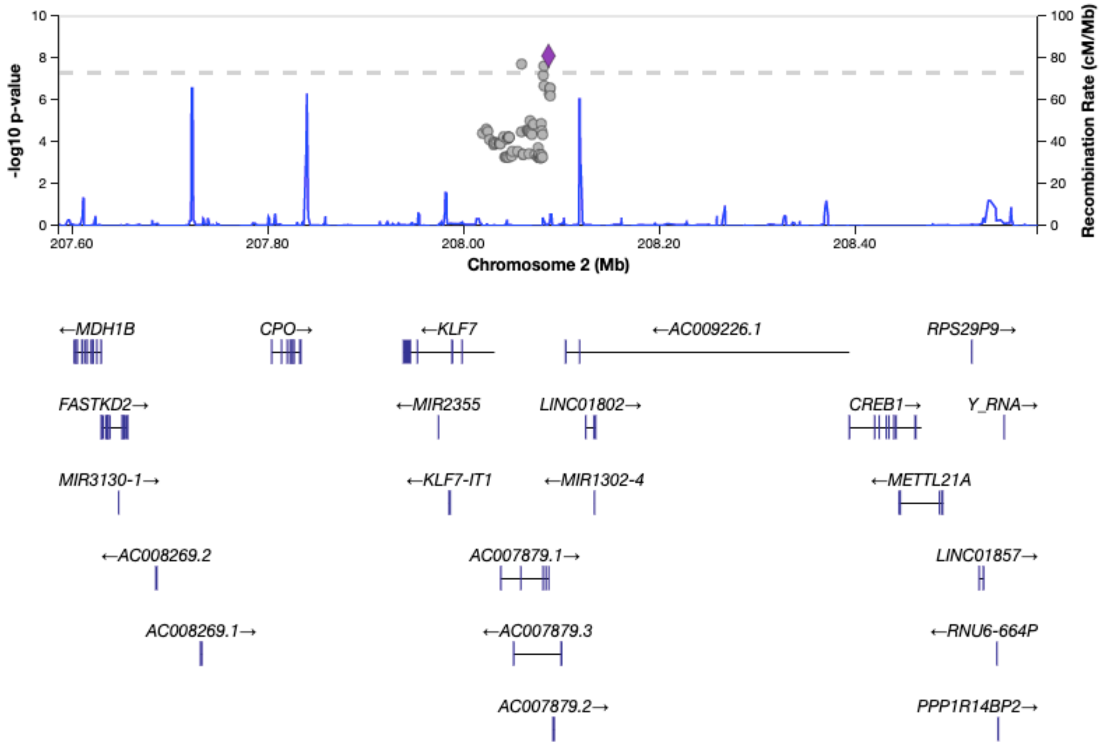

rsID: rs3915060

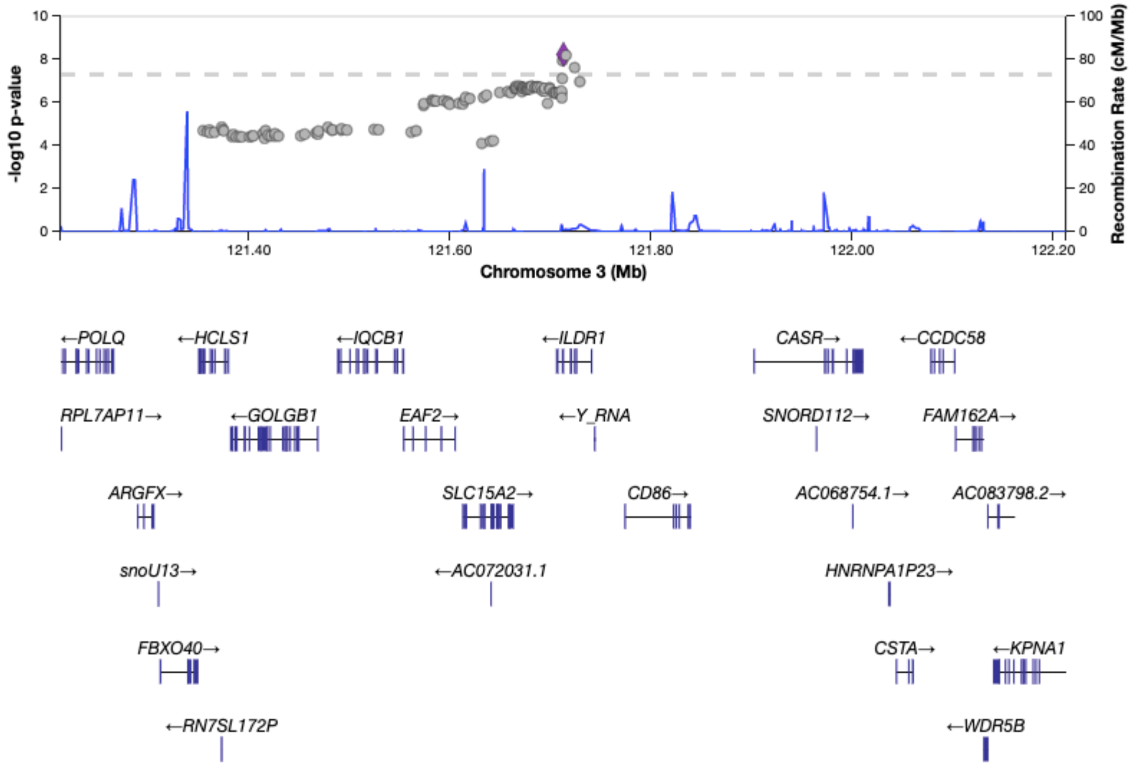

rsID: rs6443802

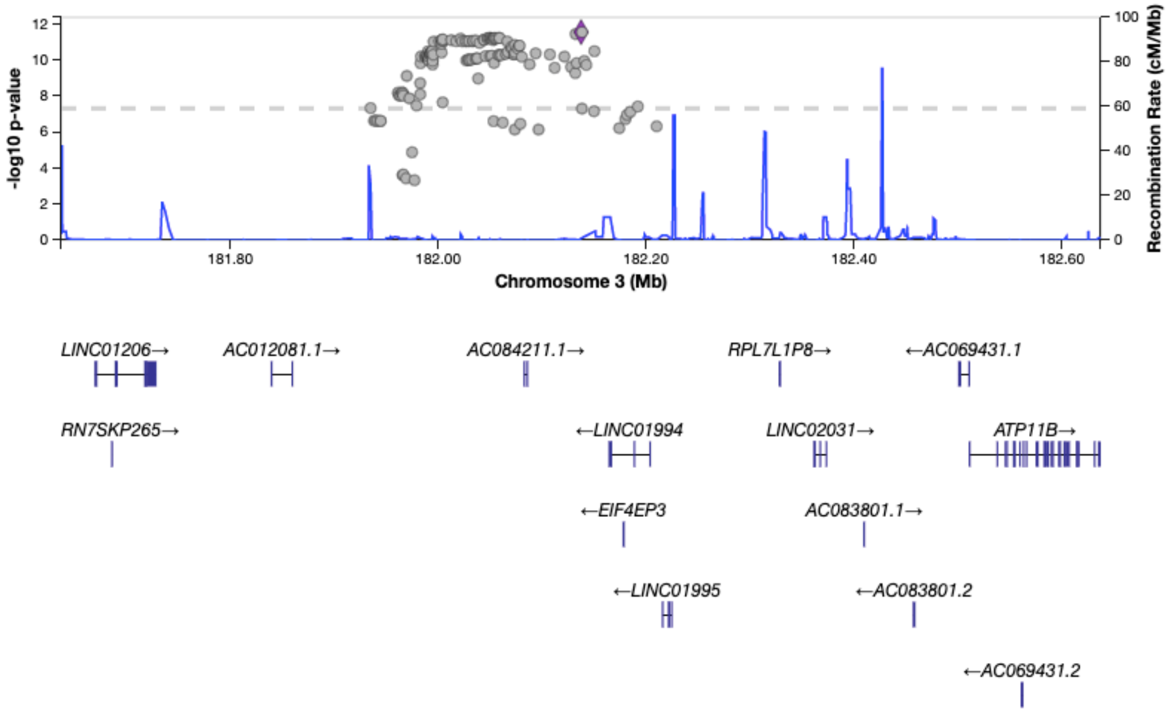

rsID: rs13148153

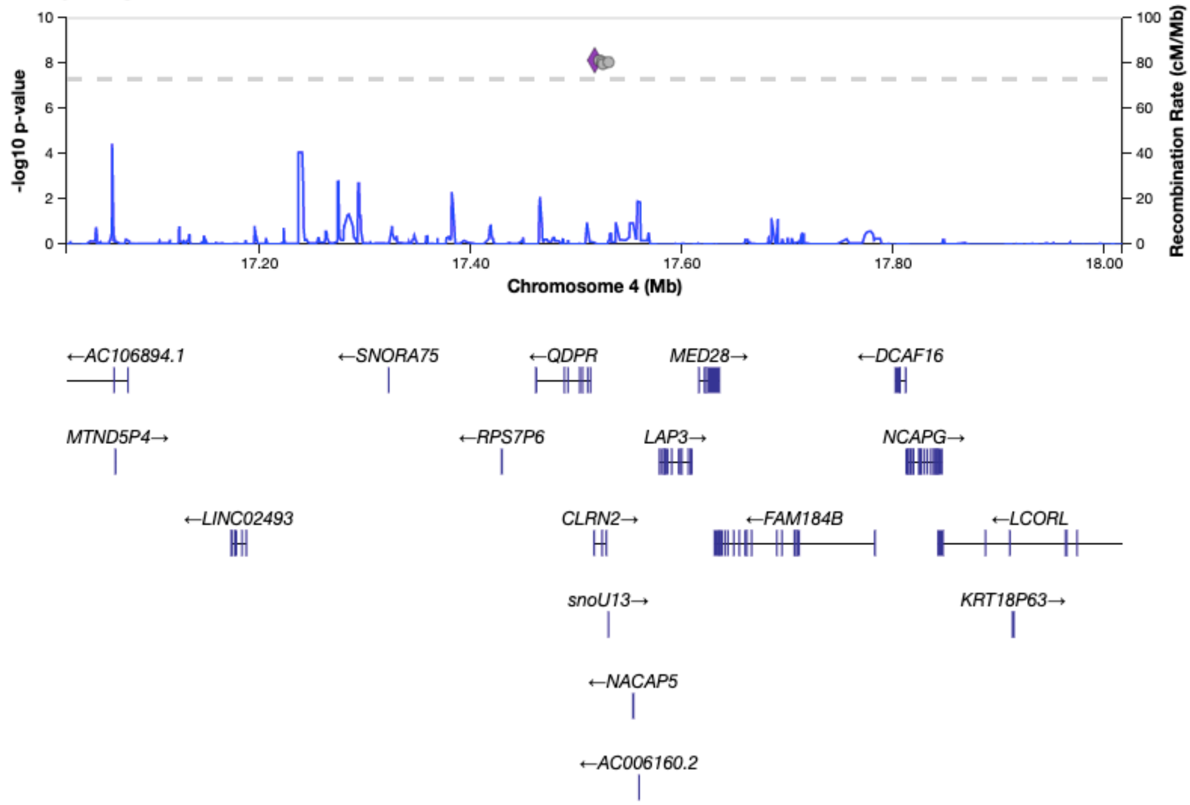

rsID: rs34929759

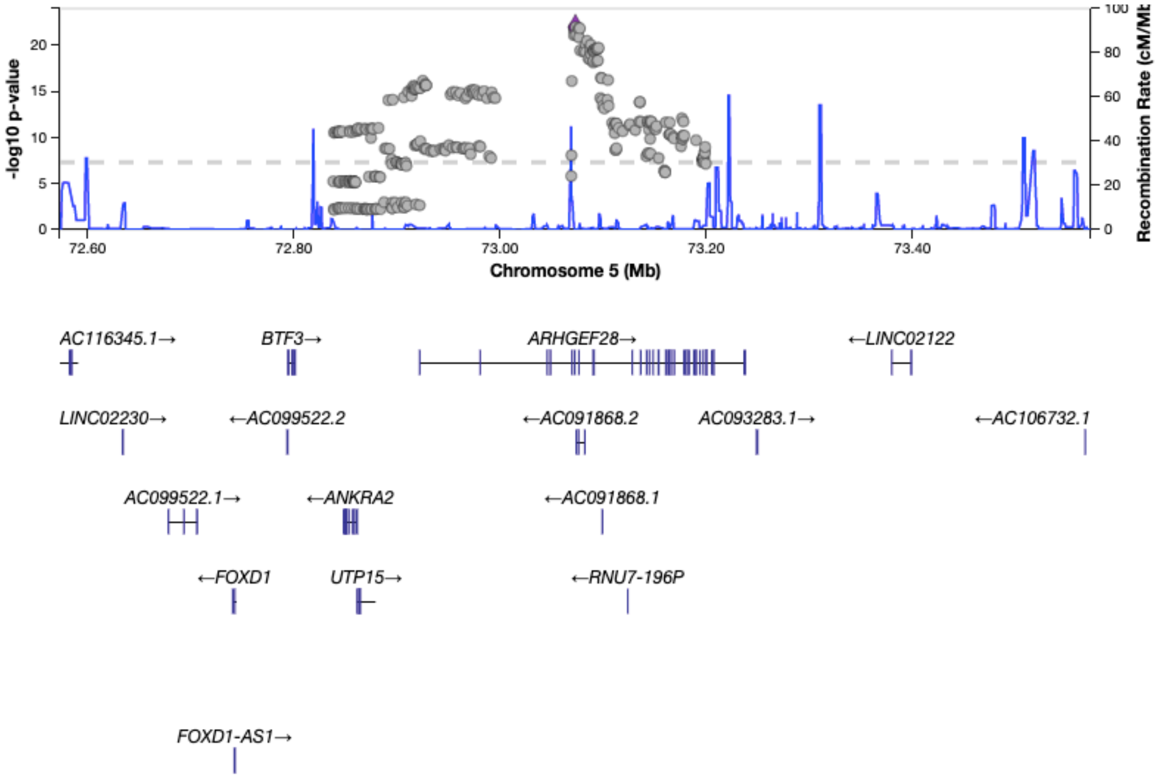

rsID: rs10948071

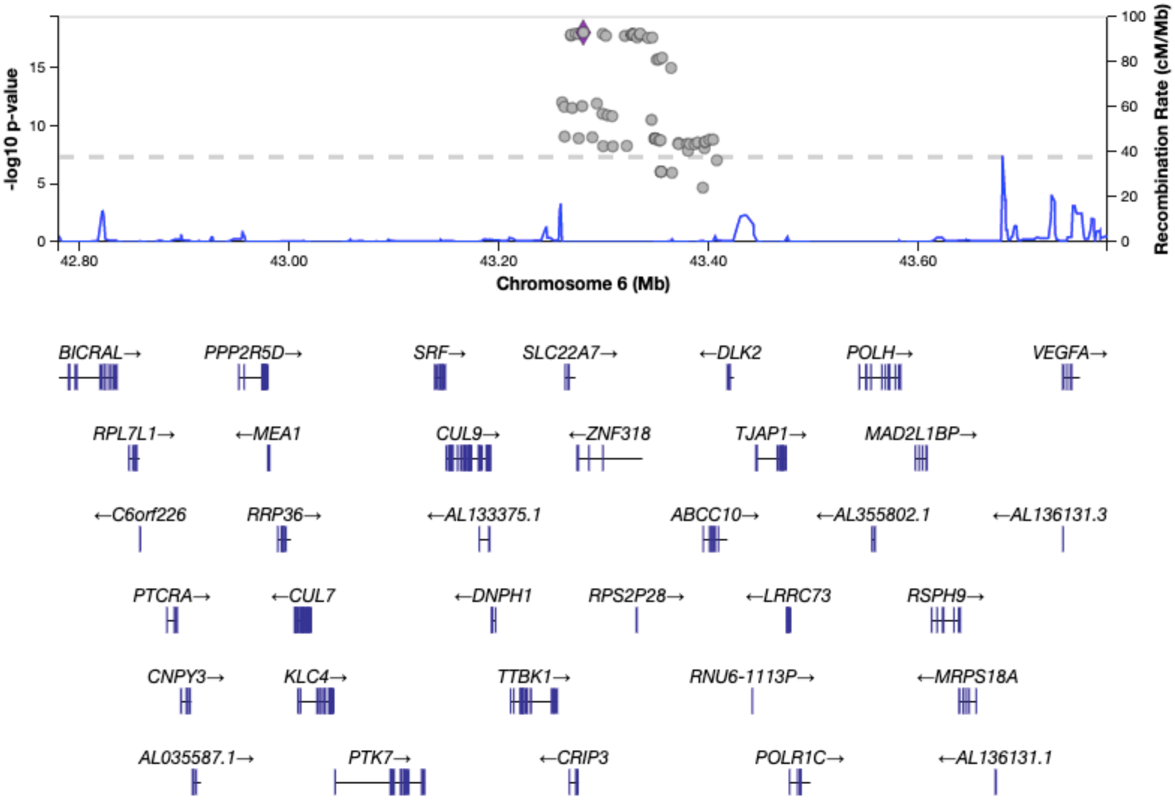

rsID: rs9493627

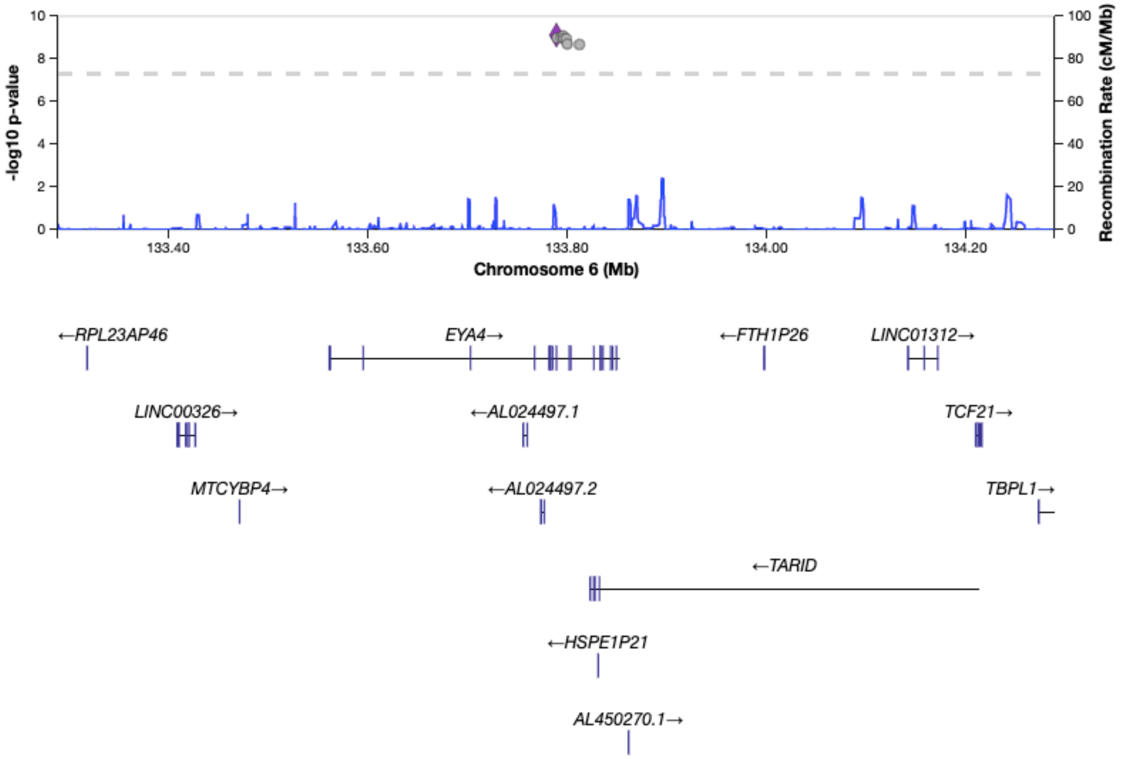

rsID: rs6902016

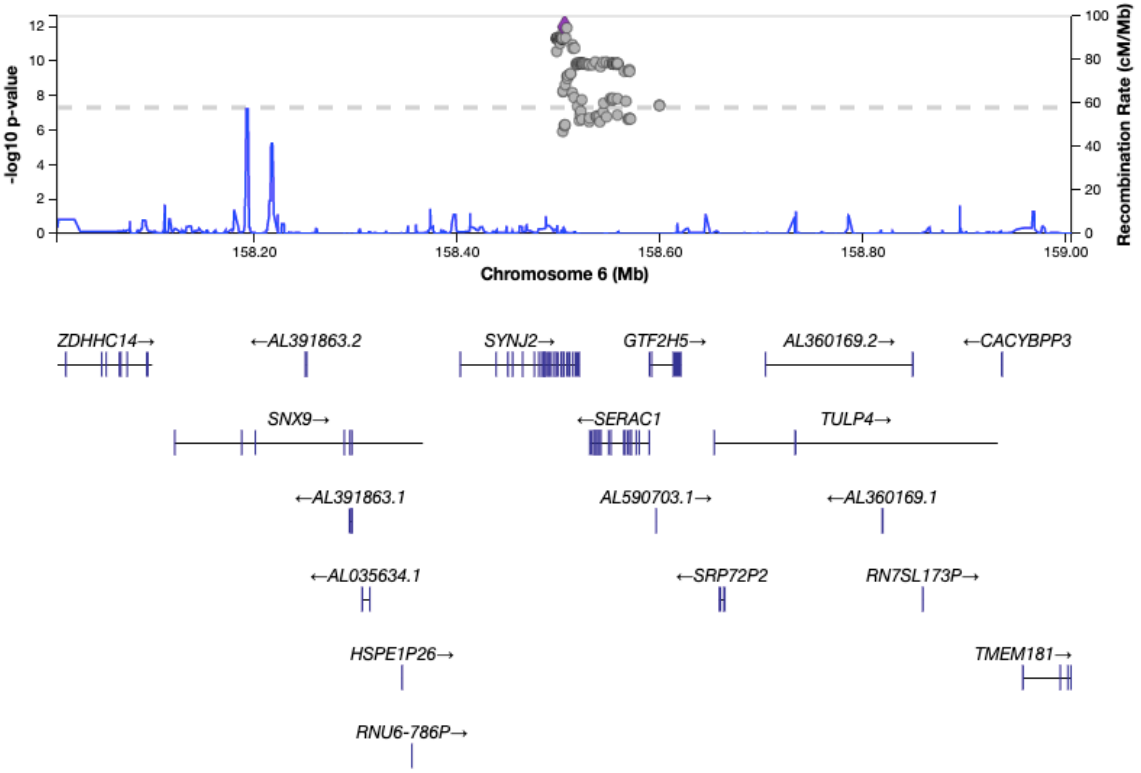

rsID: rs6968827

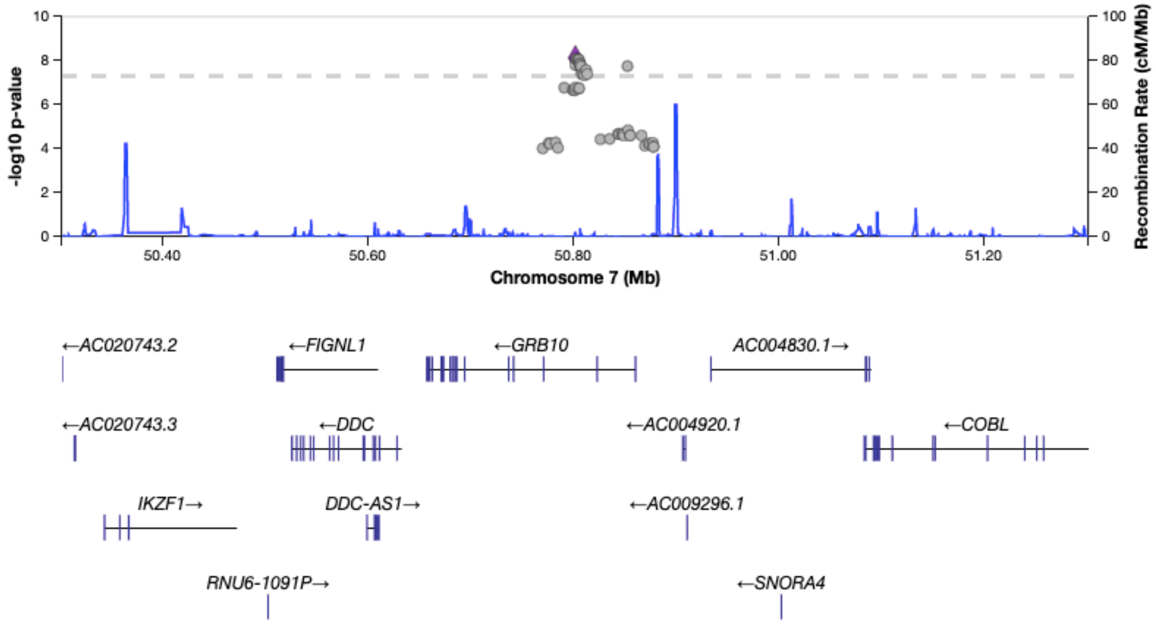

rsID: rs4732339

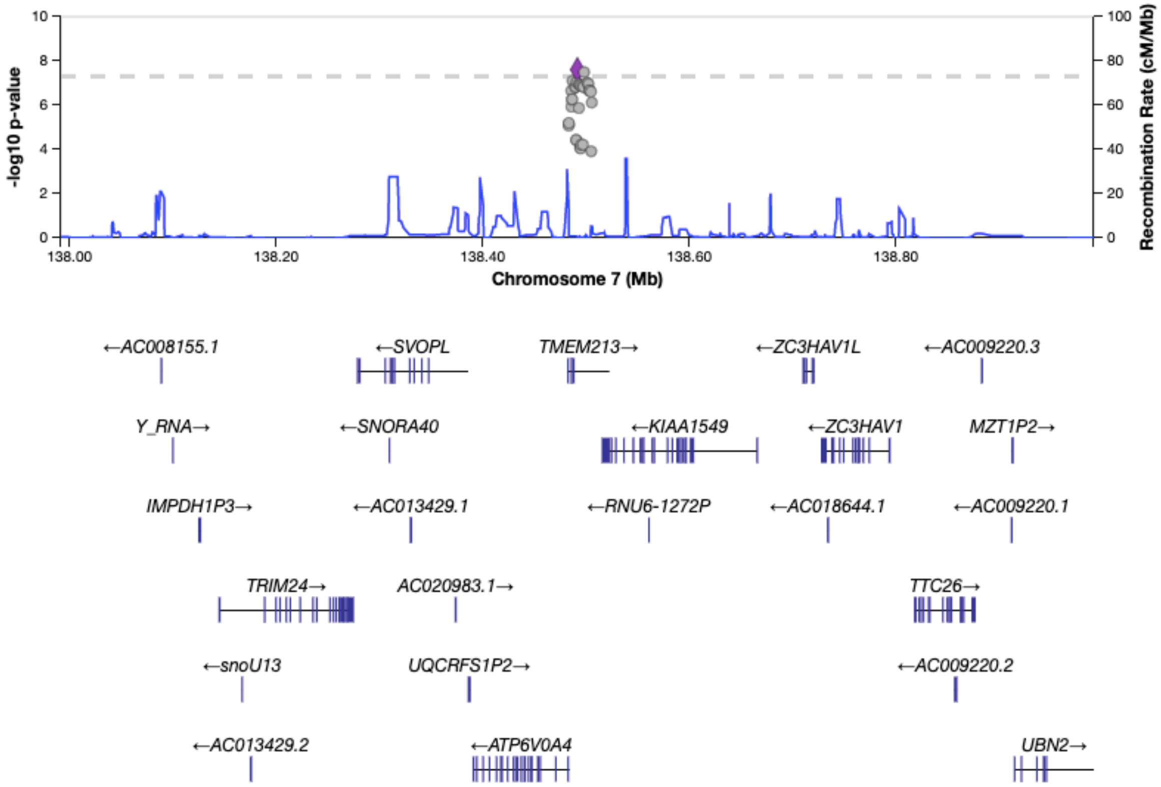

rsID: rs74544416

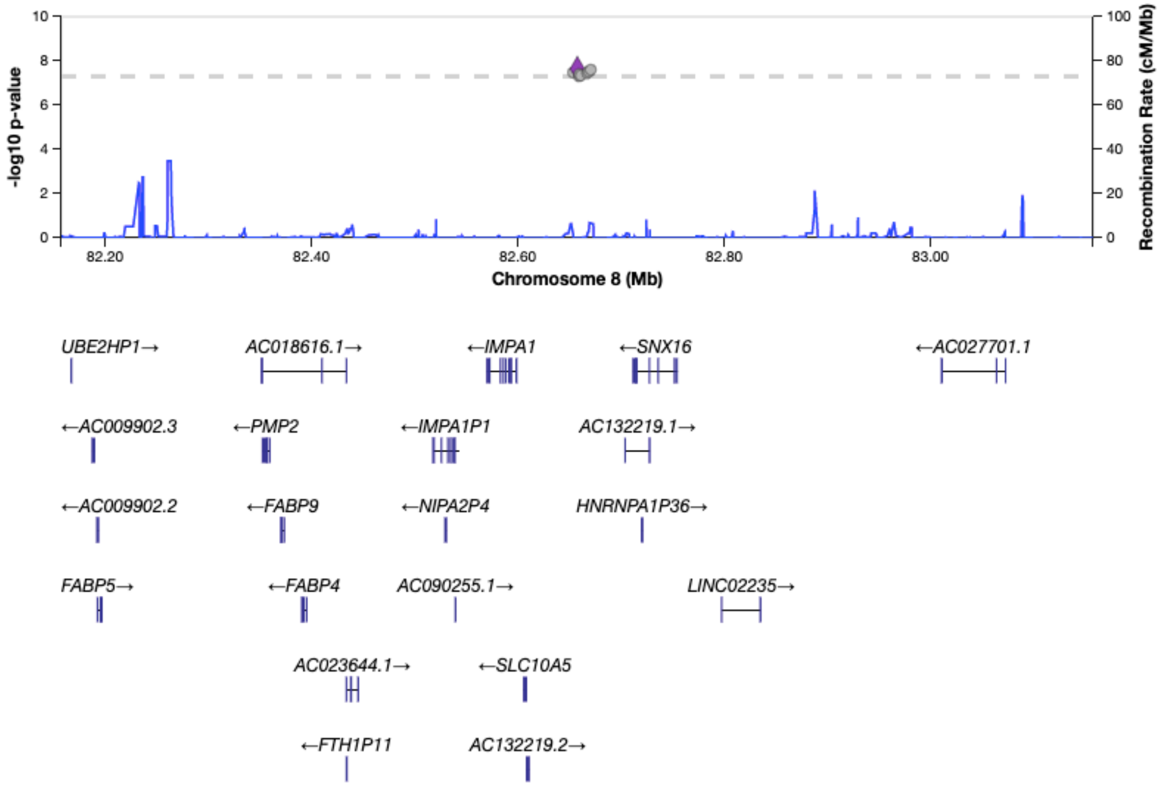

rsID: rs13277721

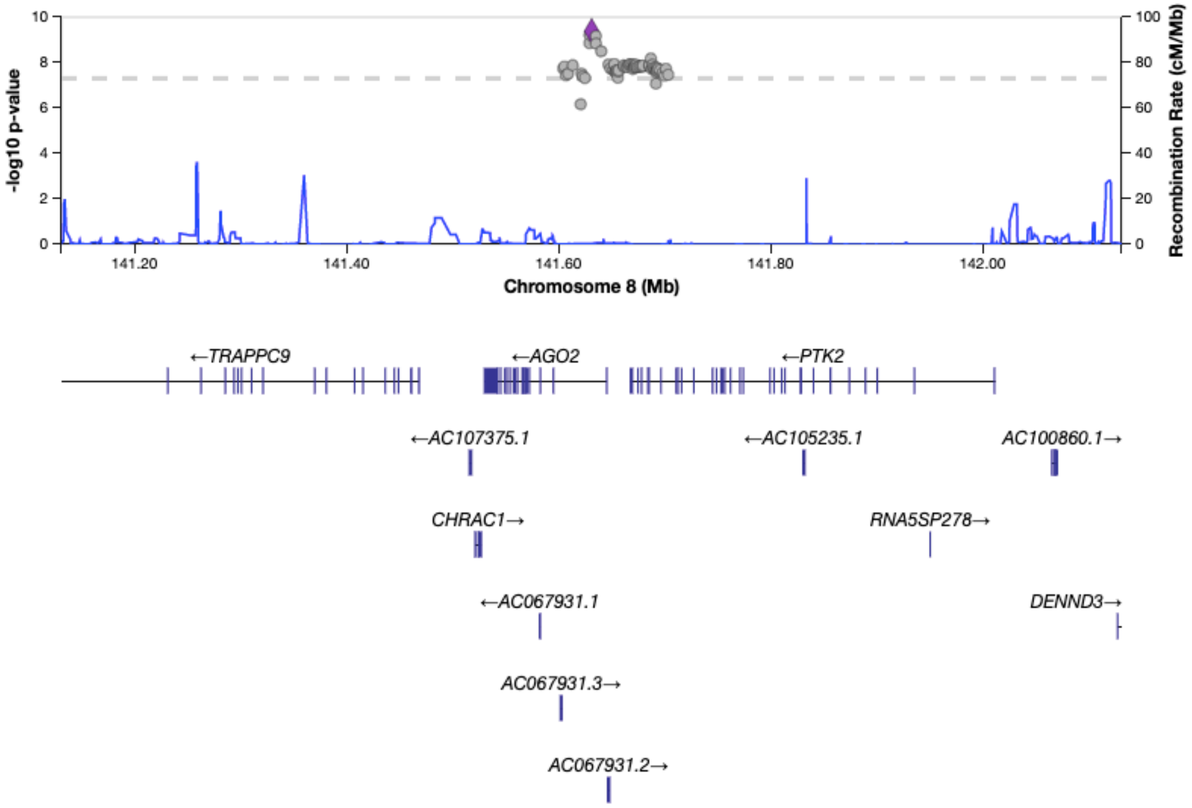

rsID: rs117583072

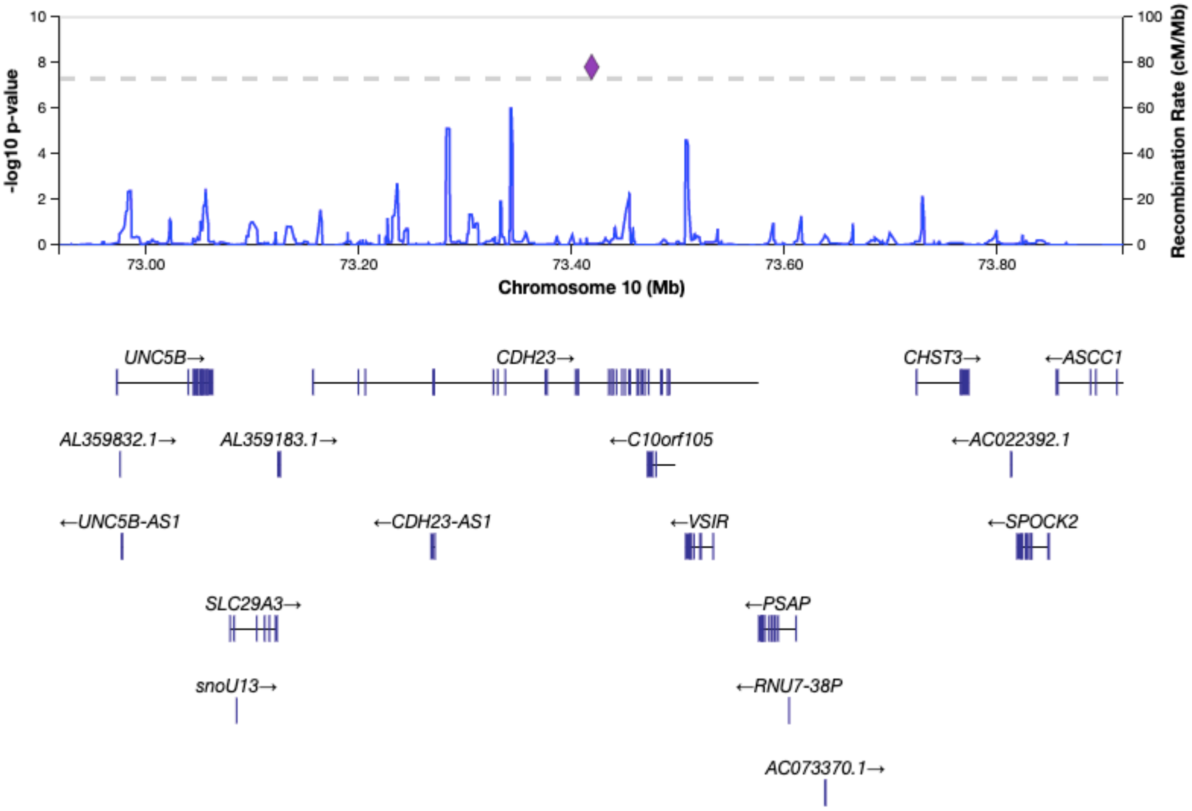

rsID: rs835259

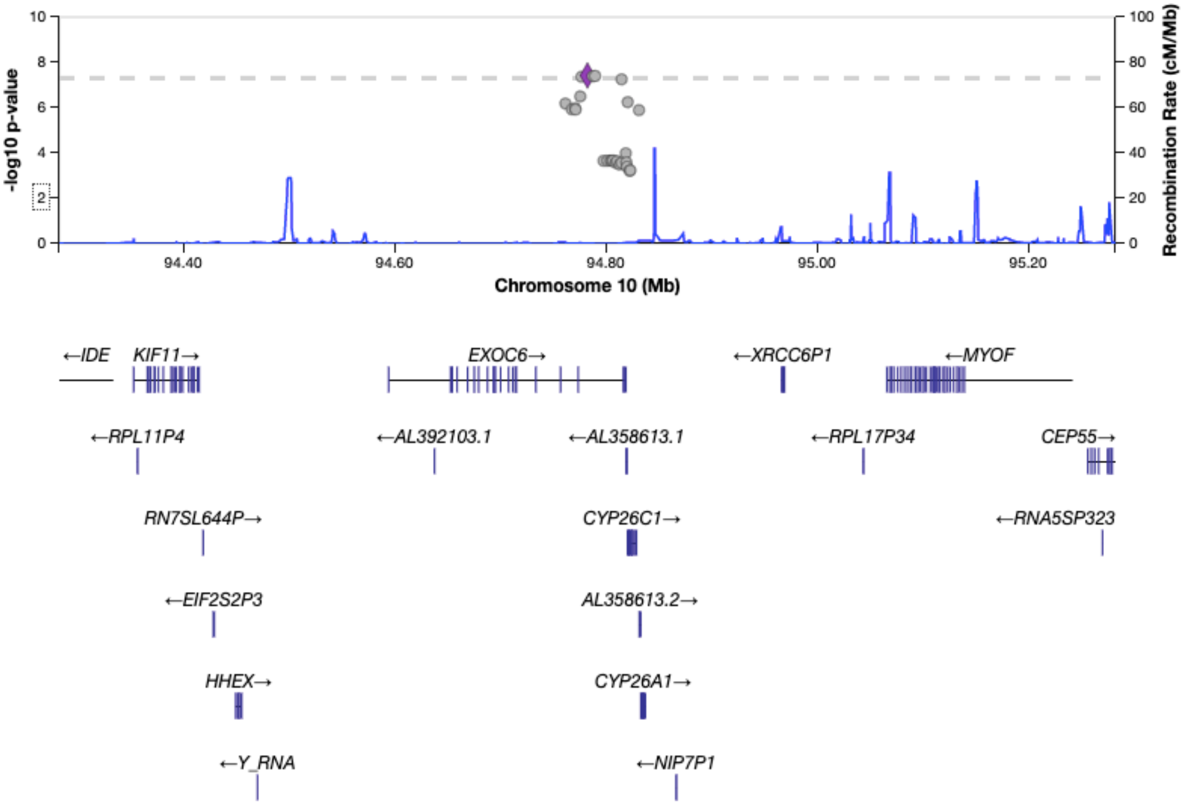

rsID: rs10901863

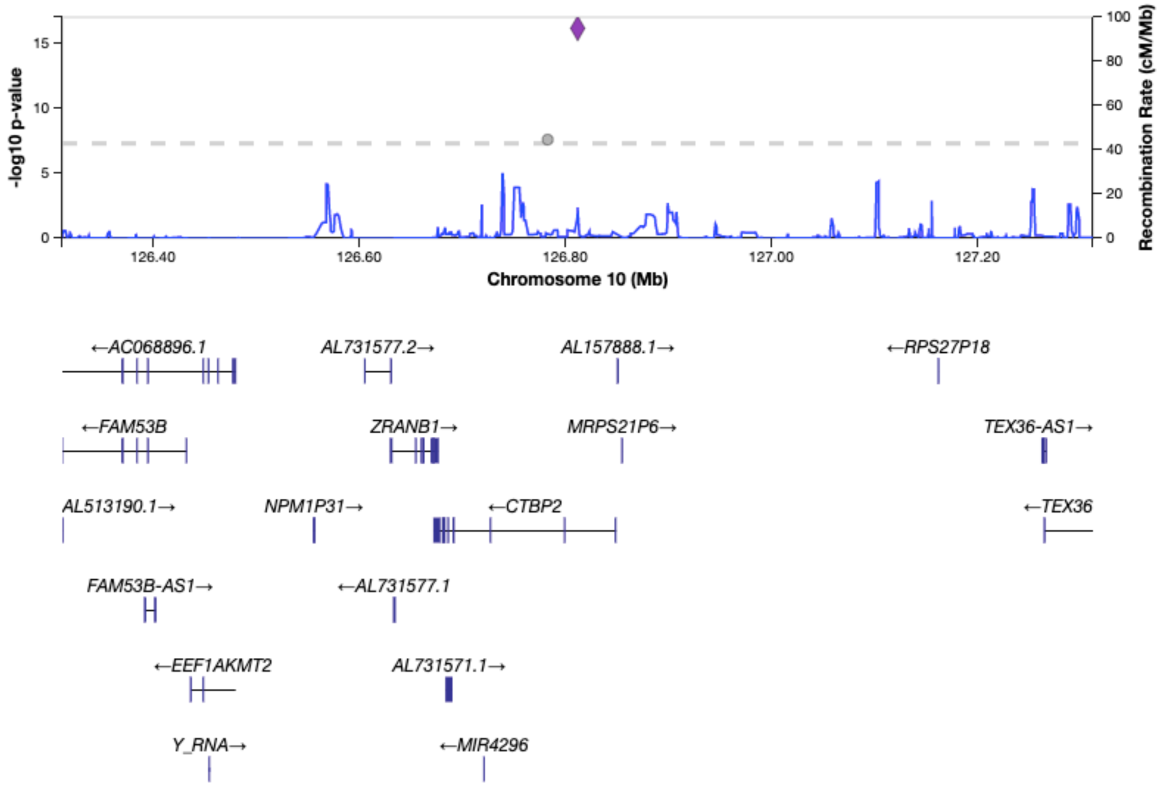

rsID: rs55635402

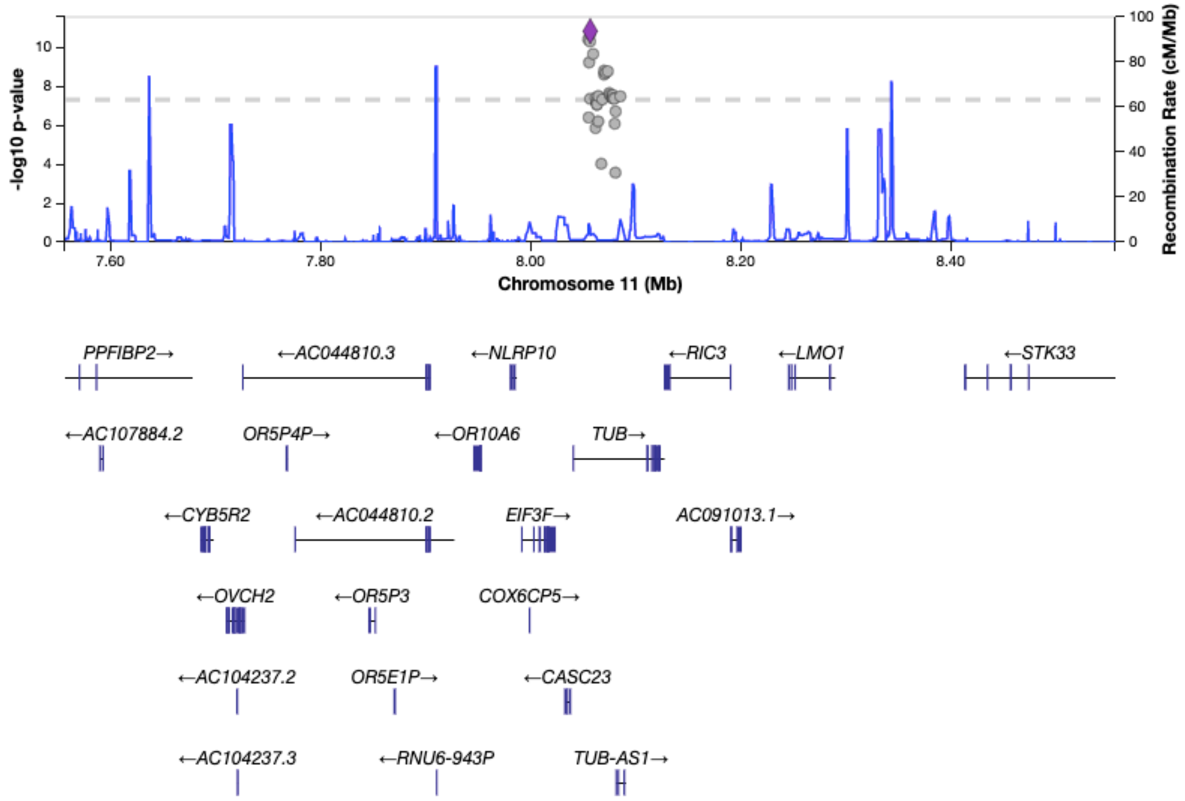

rsID: rs61890355

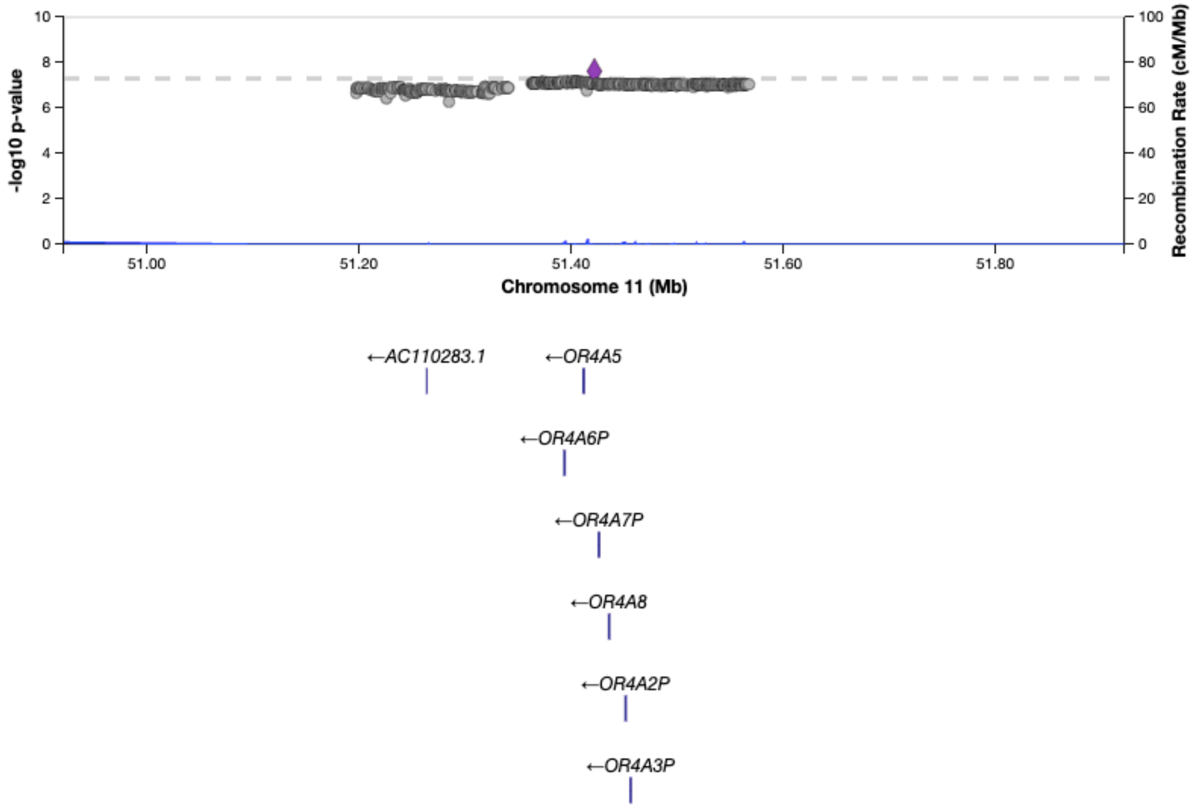

rsID: rs118176061

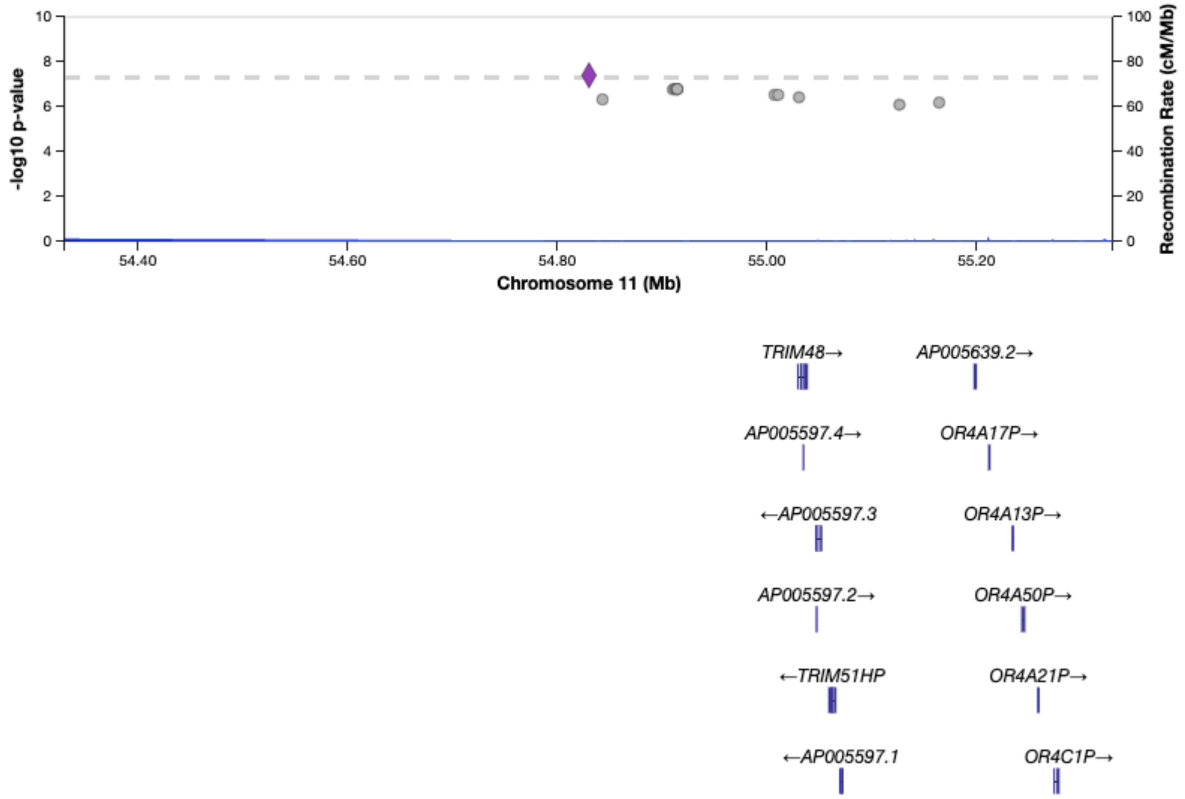

rsID: rs1126809

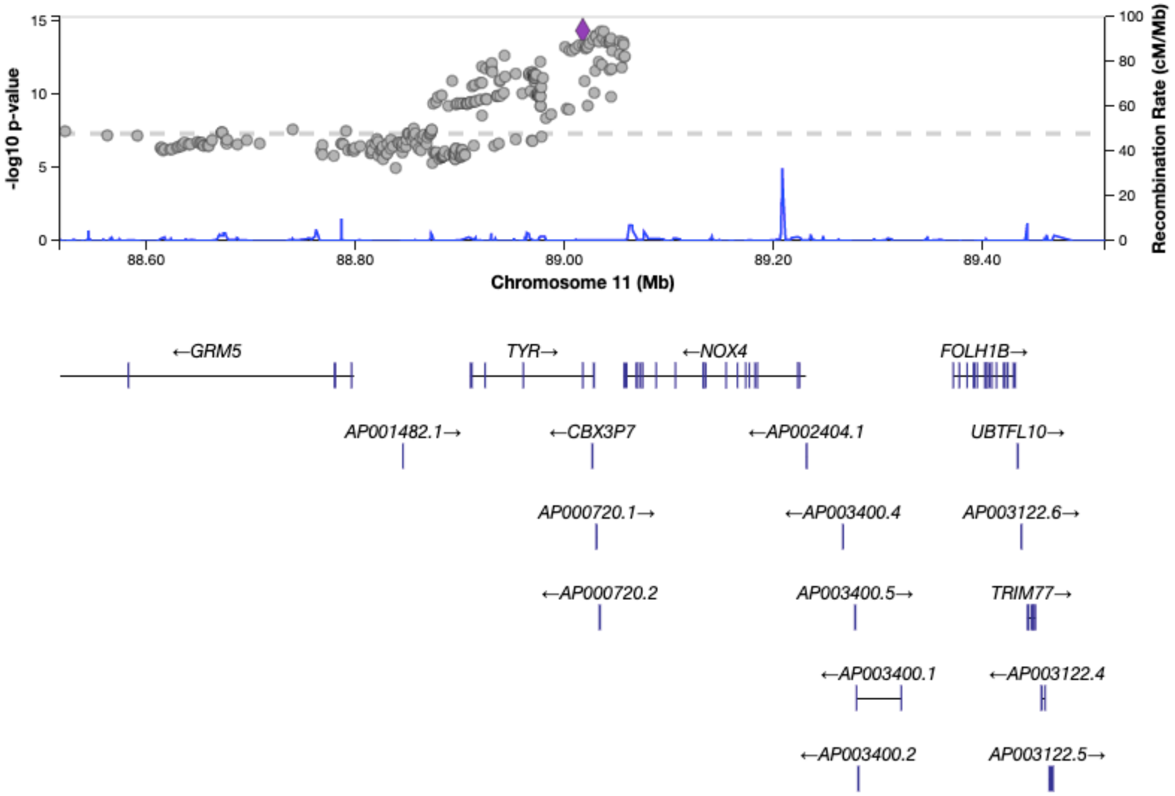

rsID: rs67307131

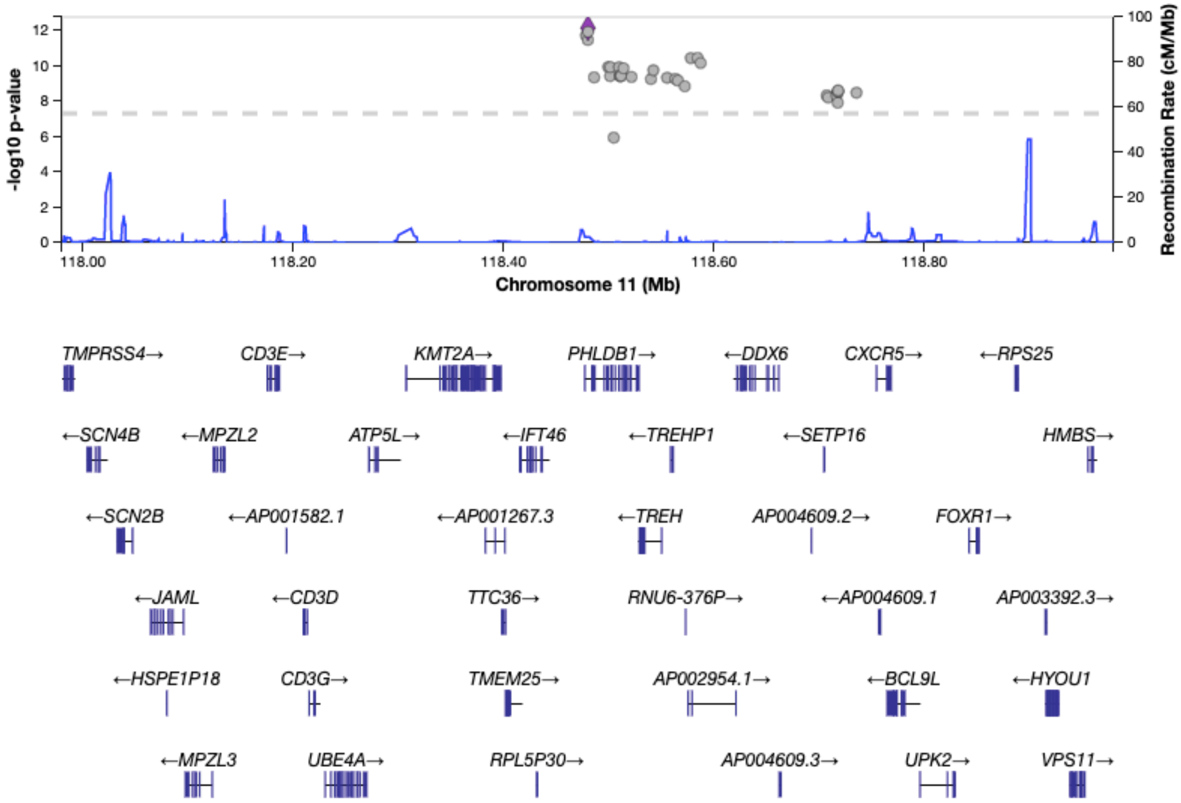

rsID: rs1566129

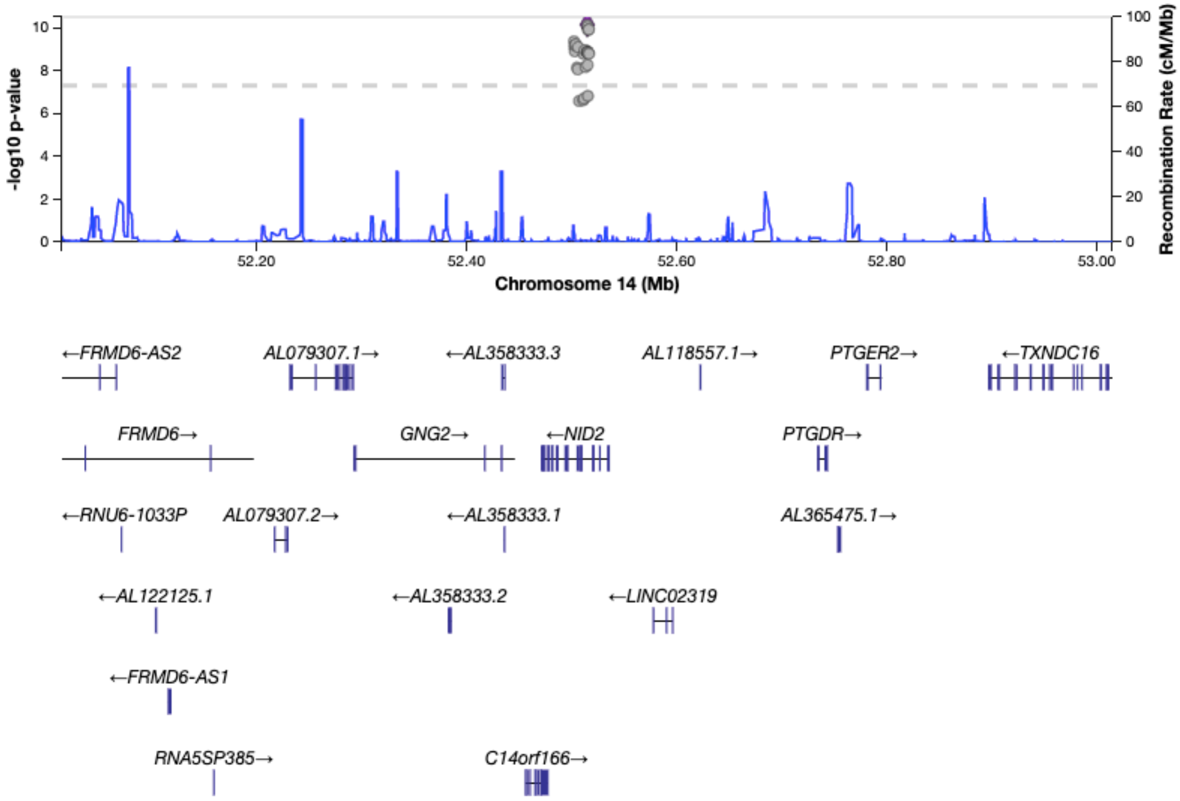

rsID: rs62033400

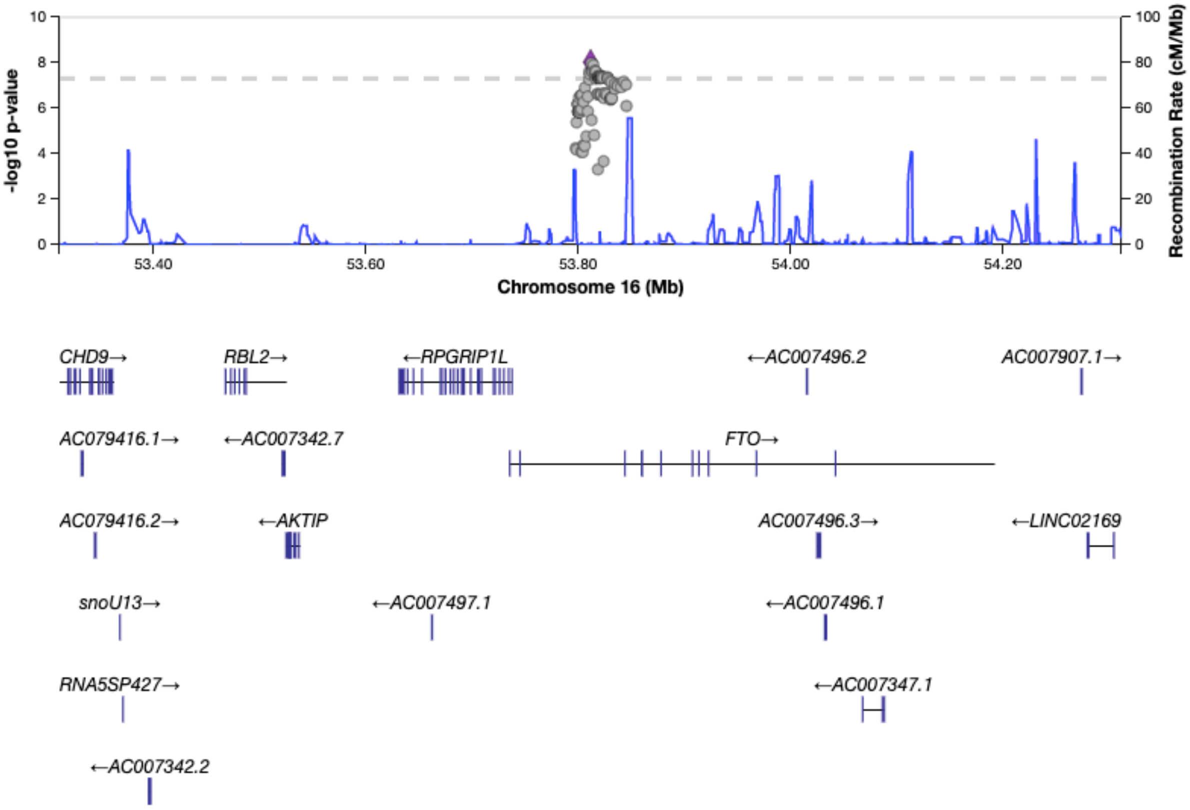

rsID: rs78417468

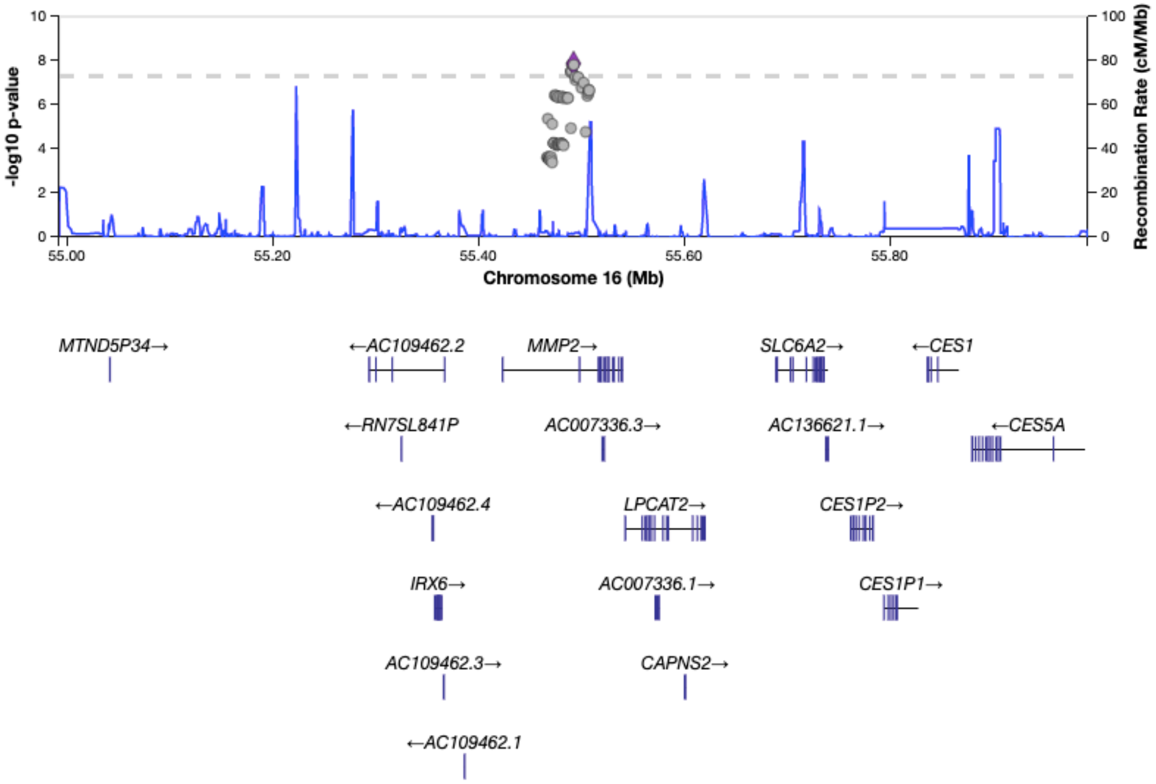

rsID: rs12938775

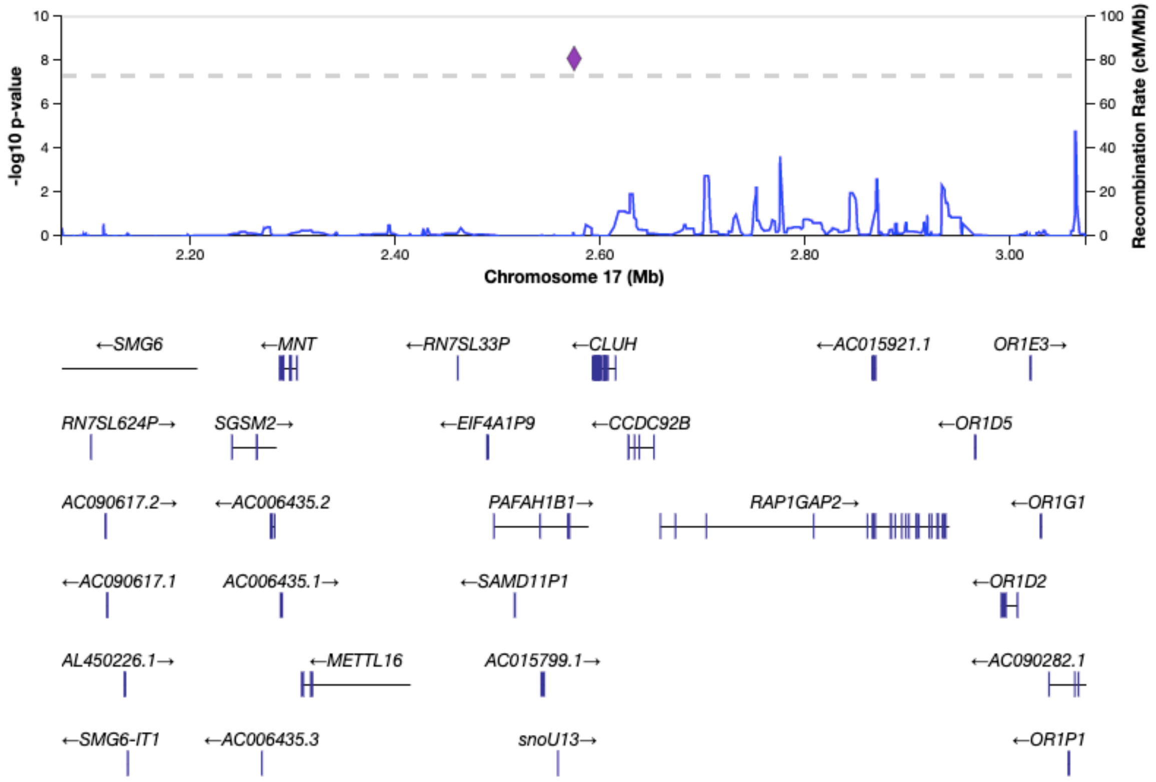

rsID: rs118174674

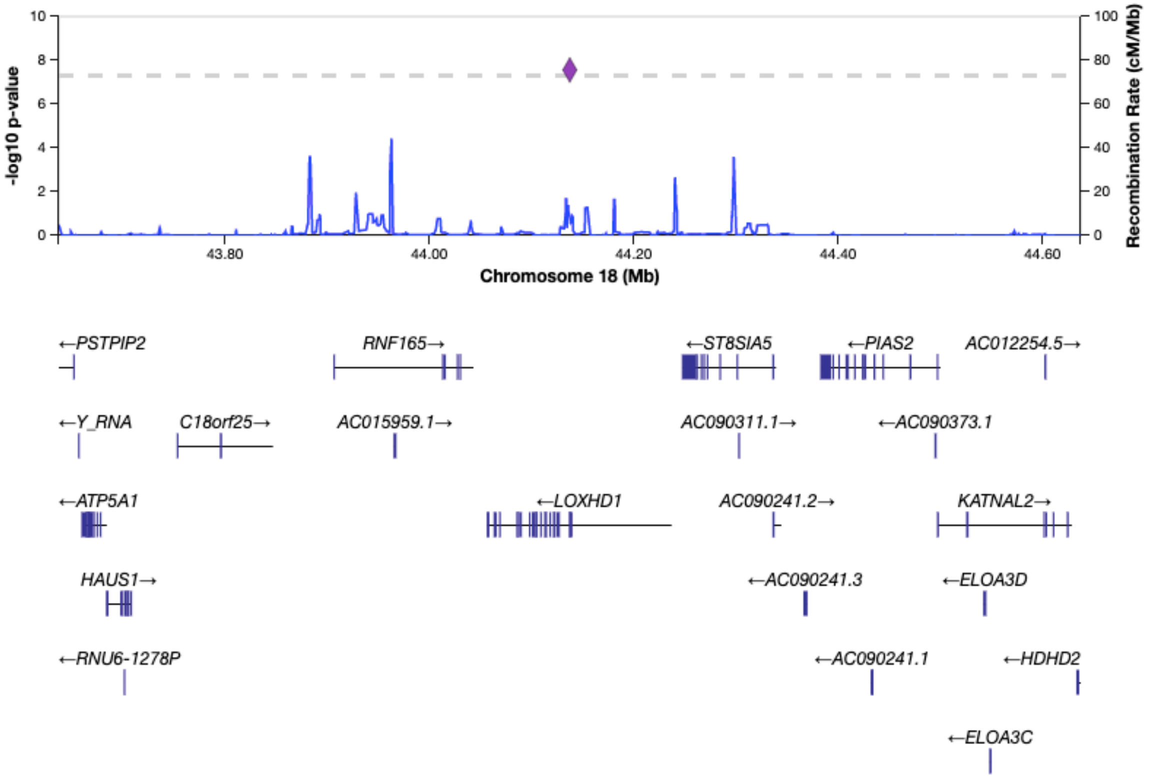

rsID: rs4611552

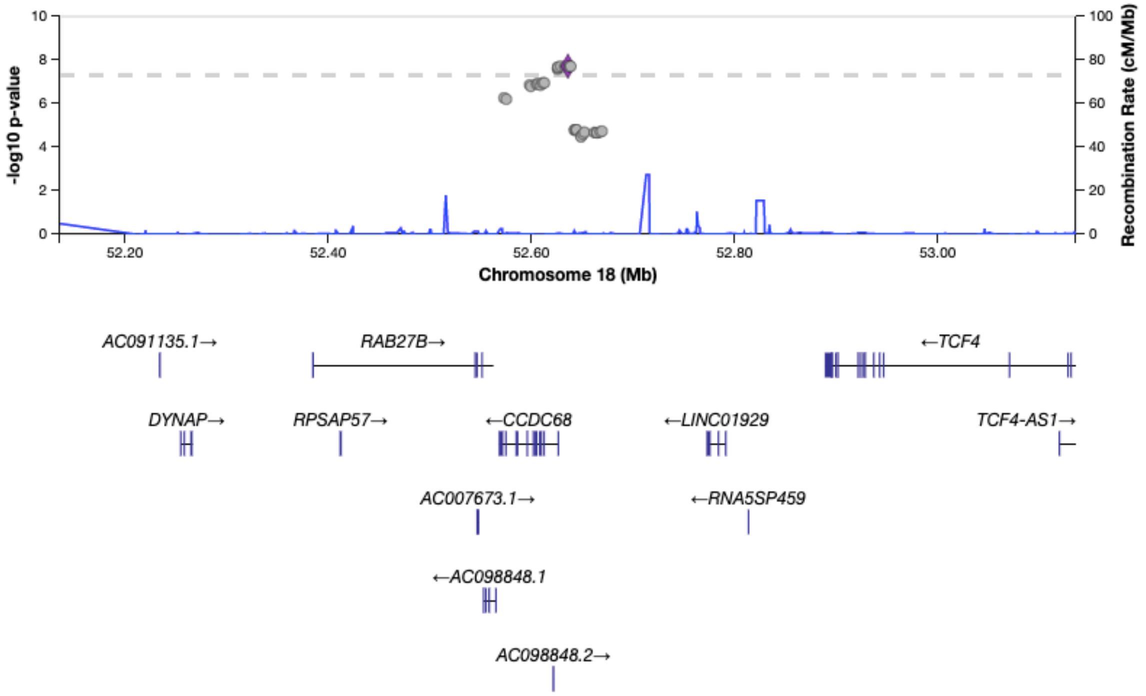

rsID: rs11881070

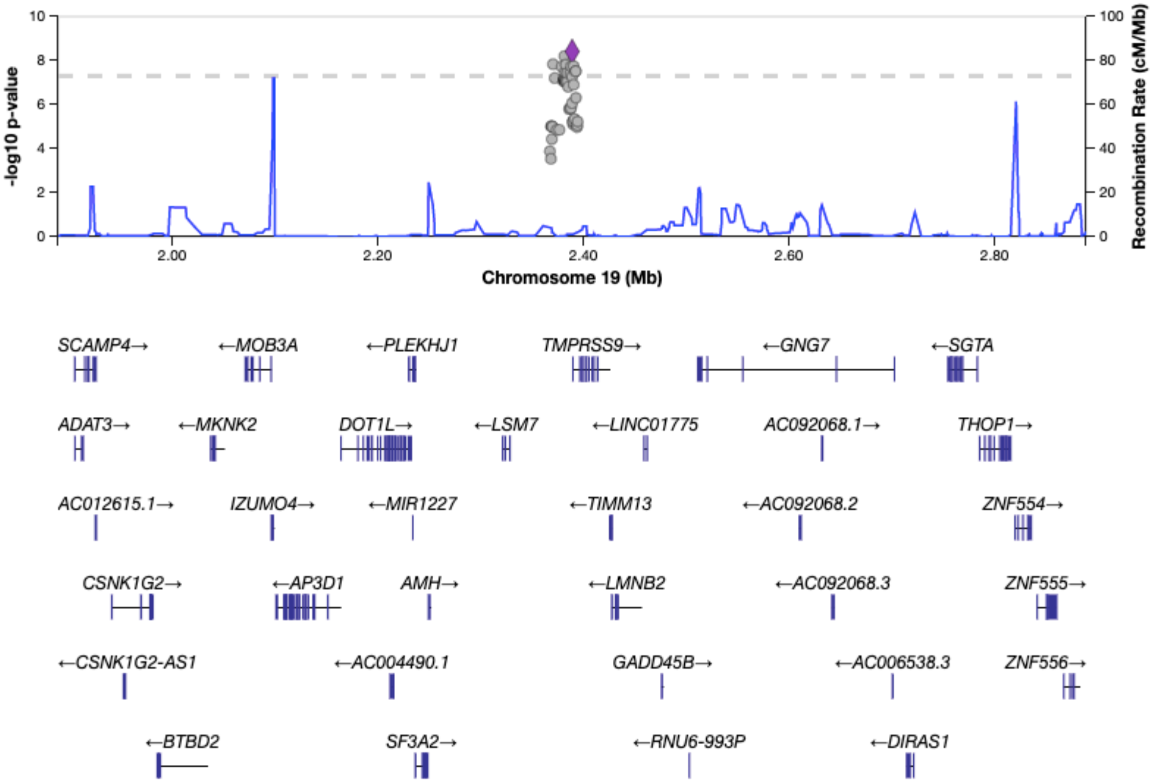

rsID: rs132929

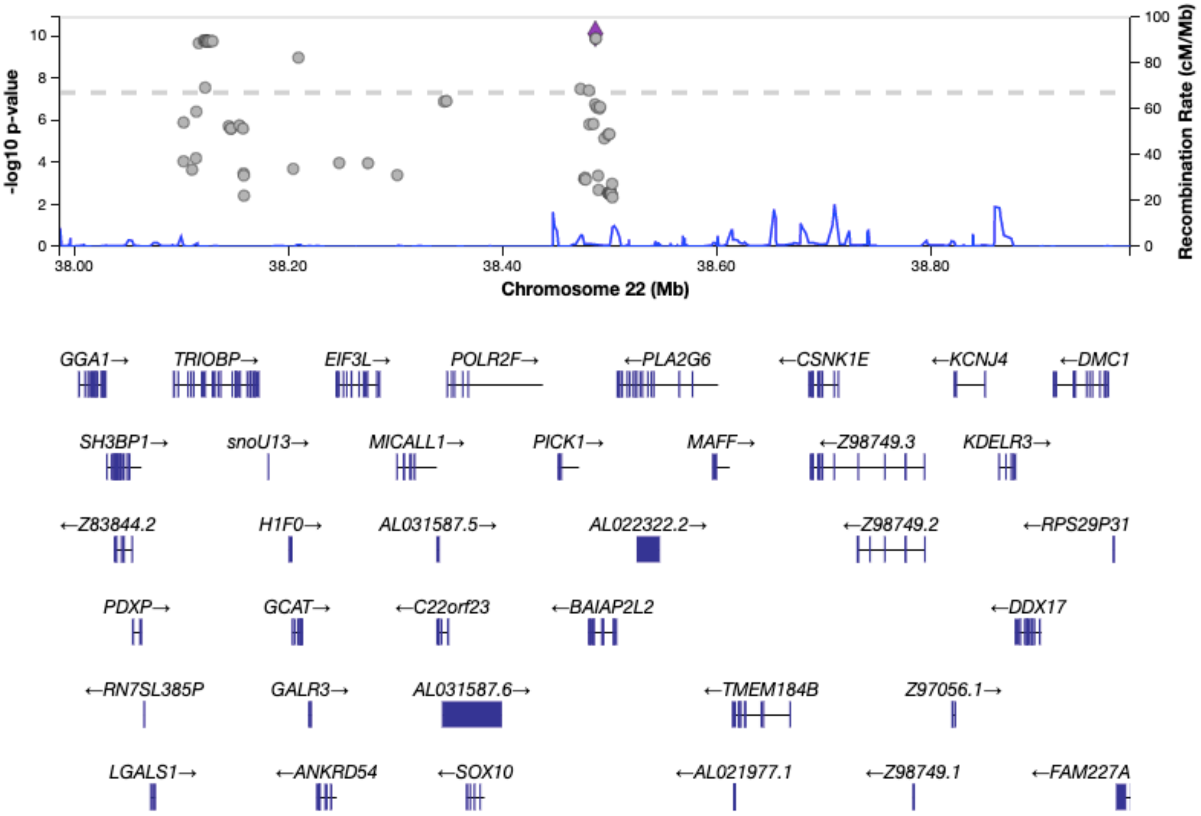

rsID: rs36062310

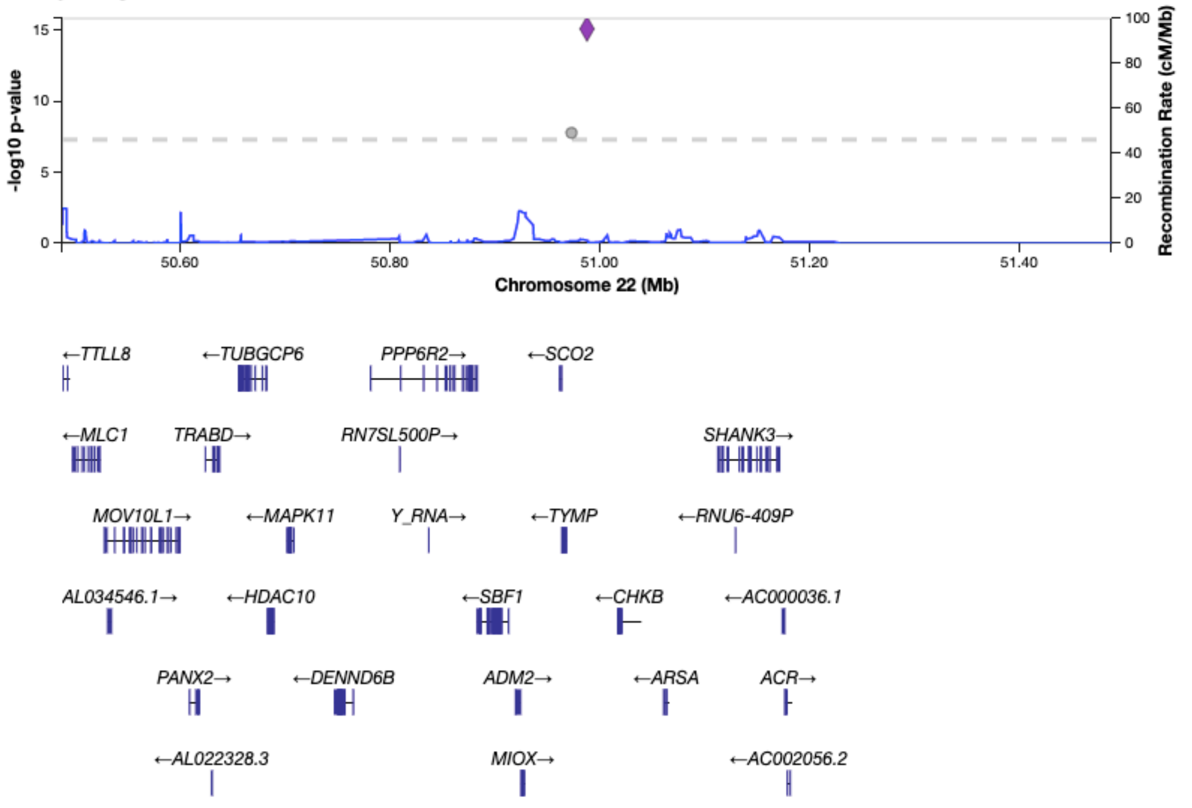

Supplement: S2 Fig — Each plot displays the -log10(p-values) and genomic locations of all SNPs that are in linkage disequilibrium (LD; r2 > 0.6) with an LD-independent, genome-wide significant SNP (p < 5e-8). Genes at and around each locus are shown below each plot. Eight novel loci that had not been reported in previous GWAS of hearing difficulty are indicated in bold. (PDF) [file pgen.1009025.s002.pdf]
