# Supplementary material for: Biological insights from multi-omic analysis of 31 genomic risk loci for adult hearing difficulty
Source: PLoS Genet. 2020 Sep 28;16(9):e1009025. doi: 10.1371/journal.pgen.1009025 (PMC7544108; doi:10.1371/journal.pgen.1009025)
Supplement: S2 Table — Genetic correlation between hearing-related traits and 234 non-hearing related traits measured in independent cohorts, using LDHub. For each pair of traits, we report the genetic correlation (rg) and its associated p-value. (PDF) [file pgen.1009025.s008.pdf]

Trait	PMID	rg.2247_1	rg.2257
Hearing difficulty	UKBB	1	0.8132
Background noise problems	UKBB	0.8132	1
Hearing aid user	UKBB	0.7584	0.3719
Tinnitus	UKBB	0.5816	0.4385
Neuroticism	27089181	0.2132	0.3756
Depressive symptoms	27089181	0.2991	0.3857
Subjective well being	27089181	-0.2432	-0.4206
PGC cross-disorder analysis	23453885	0.2357	0.3496
Schizophrenia	25056061	0.0718	0.2151
Neuroticism	24828478	0.2157	0.4294
Autism spectrum disorder	0	0.1206	0.2568
Insomnia	28604731	0.2796	0.2447
Age of first birth	27798627	-0.1656	-0.1592
Insomnia	27992416	0.2401	0.2154
Major depressive disorder	22472876	0.2046	0.2529
Waist circumference	25673412	0.132	0.0662
Excessive daytime sleepiness	27992416	0.1397	0.1979
Obesity class 1	23563607	0.1347	0.0601
Waist-to-hip ratio	25673412	0.1308	0.0629
Body fat	26833246	0.1529	0.0697
Bipolar disorder	21926972	0.1093	0.1588
College completion	23722424	-0.1075	-0.0259
Chronotype	27494321	-0.1281	-0.12
Number of children ever born	27798627	0.1526	0.1169
Overweight	23563607	0.1246	0.07
Neo-conscientiousness	21173776	-0.3849	-0.2974
Years of schooling (proxy cognitive performance)	25201988	-0.1416	-0.0291
Cigarettes smoked per day	20418890	0.3149	0.1919
Neo-openness to experience	21173776	0.2439	0.3039
Leptin_not_adjBMI	26833098	0.2205	0.0376
Ever vs never smoked	20418890	0.1554	0.0606
Forced expiratory volume in 1 second (FEV1)/Forced Vital capacity(	28166213	-2.00E-04	0.0204
Obesity class 2	23563607	0.1262	0.0408
HDL cholesterol	20686565	-0.0669	-0.0133
Years of schooling 2013	23722424	-0.1253	-0.0105
Ratio of bisallylic groups to double bonds	27005778	-0.0683	-0.009
Mothers age at death	27015805	-0.1143	-0.052
Ratio of bisallylic groups to total fatty acids	27005778	-0.0462	0.0237
Body mass index	20935630	0.096	0.063
Childhood obesity	22484627	0.0506	0.0182
Hip circumference	25673412	0.0941	0.0494
Attention deficit hyperactivity disorder	20732625	0.3019	0.213
Forced expiratory volume in 1 second (FEV1)	26635082	-0.013	0.0451
Forced Vital capacity(FVC)	26635082	0.0123	0.0551

Obesity class 3	23563607	0.1492	0.0756
Years of schooling 2016	27225129	-0.0363	0.0145
Attention deficit hyperactivity disorder (GC)	27663945	0.2859	0.1767
Attention deficit hyperactivity disorder (No GC)	27663945	0.2862	0.1772
Triglycerides	20686565	0.0536	0.029
Average number of double bonds in a fatty acid chain	27005778	-0.0207	0.0659
Forced expiratory volume in 1 second (FEV1)	21946350	-0.1688	0.0406
Forced expiratory volume in 1 second (FEV1)	28166213	-0.0066	0.0381
Average number of methylene groups per a double bond	27005778	0.0322	-0.0363
Former vs Current smoker	20418890	-0.1698	-0.022
Glutamine	27005778	-0.2094	-0.0783
Total lipids in small HDL	27005778	0.1742	0.0279
Rheumatoid Arthritis	24390342	0.09	0.0102
Extreme bmi	23563607	0.1005	0.0297
ICV	25607358	0.0277	0.0397
Leptin_adjBMI	26833098	0.1496	-0.059
Urinary albumin-to-creatinine ratio	26631737	-0.1069	-0.1017
Extreme height	23563607	-0.0487	-0.0503
Forced expiratory volume in 1 second (FEV1)/Forced Vital capacity(	21946350	-0.1056	-0.0283
Age at Menarche	25231870	-0.0432	-0.0593
Age at Menopause	26414677	-0.0403	-0.077
Age of smoking initiation	20418890	-0.1775	-0.0486
Primary sclerosing cholangitis	27992413	-0.0666	-0.1027
Height_2010	20881960	-0.0516	-0.0253
Forced expiratory volume in 1 second (FEV1)/Forced Vital capacity(	26635082	-0.0658	-0.041
22:6 docosahexaenoic acid	27005778	-0.1373	0.0165
Extreme waist-to-hip ratio	23563607	0.0759	0.0024
Phospholipids in medium LDL	27005778	0.1608	0.1672
Concentration of small LDL particles	27005778	0.1239	0.146
Total cholesterol in medium LDL	27005778	0.1785	0.184
Urinary albumin-to-creatinine ratio (non-diabetes)	26631737	-0.0906	-0.1047
Systemic lupus erythematosus	26502338	-0.0411	-0.0897
Free cholesterol	27005778	0.1398	0.2466
Mean Caudate	25607358	-0.0105	0.004
Creatinine	27005778	-0.0976	-0.0539
Omega-3 fatty acids	27005778	-0.1017	0.039
Total lipids in small LDL	27005778	0.1294	0.1479
Difference in height between adolescence and adulthood; age 14	23449627	-0.1136	-0.1332
Total cholesterol in medium HDL	27005778	0.0731	-0.0102
Triglycerides in very large HDL	27005778	0.0916	0.1253
Forced Vital capacity(FVC)	28166213	-0.0113	0.037
Multiple sclerosis	21833088	0.1062	0.161
Ulcerative colitis	26192919	0.0474	0.0521
Amyotrophic lateral sclerosis	27455348	-0.0621	-0.1275
Serum creatinine (non-diabetes)	26831199	0.0151	0.0295

Sitting height ratio	25865494	-0.0957	0.0241
Total cholesterol in LDL	27005778	0.1647	0.1672
Infant head circumference	22504419	-0.112	-0.0838
Triglycerides in small HDL	27005778	0.1655	0.0282
Total cholesterol in small LDL	27005778	0.1376	0.1546
Mean Hippocampus	25607358	-0.0184	-0.1253
Concentration of medium LDL particles	27005778	0.1303	0.147
Triglycerides in IDL	27005778	0.1422	0.1027
Total lipids in medium LDL	27005778	0.1298	0.1462
Anorexia Nervosa	24514567	-0.0437	0.0163
Chronic Kidney Disease	26831199	-0.1434	-0.054
Cholesterol esters in medium LDL	27005778	0.1289	0.1457
Cholesterol esters in medium HDL	27005778	0.0263	-0.0053
Free cholesterol in medium HDL	27005778	0.091	0.0164
Alanine	27005778	0.0917	0.0223
Citrate	27005778	-0.01	-0.0083
Mean diameter for HDL particles	27005778	-0.0557	0.0281
Concentration of medium HDL particles	27005778	0.0993	0.0037
Cholesterol esters in large LDL	27005778	0.1156	0.1465
Total lipids in medium HDL	27005778	0.0841	-0.0012
Serum total cholesterol	27005778	0.1074	0.1414
Concentration of large LDL particles	27005778	0.1272	0.1429
Phospholipids in medium HDL	27005778	0.1154	0.0131
Total lipids in large LDL	27005778	0.1216	0.1437
Albumin	27005778	-0.1236	-0.139
Triglycerides in very small VLDL	27005778	0.1106	0.0678
Free cholesterol to esterified cholesterol ratio	27005778	0.0465	0.2032
Lumbar Spine bone mineral density	26367794	-0.059	-0.064
Total cholesterol in HDL	27005778	0.0122	0.0498
HbA1C	20858683	0.0524	-0.0376
Total cholesterol in large LDL	27005778	0.1322	0.1416
Coronary artery disease	26343387	0.0526	0.0236
Height; Females at age 10 and males at age 12	23449627	-0.0572	-0.071
Adiponectin	22479202	-0.046	0.0478
Total cholesterol in large HDL	27005778	-0.0215	0.0415
Serum creatinine	26831199	0.0376	0.0371
Intelligence	28530673	0.0258	0.0262
Child birth length	25281659	-0.0884	-0.0529
Apolipoprotein B	27005778	0.0635	0.1155
Ferritin	25352340	0.0475	0.0154
Total cholesterol in IDL	27005778	0.0824	0.135
Acetoacetate	27005778	-0.0431	-0.0406
Valine	27005778	0.0117	0.0148
18:2 linoleic acid (LA)	27005778	0.0661	0.1087
Total lipids in IDL	27005778	0.0991	0.1254

Childhood IQ	23358156	-0.0842	-0.0497
Phospholipids in large LDL	27005778	0.1342	0.126
Parkinsons disease	19915575	0.0717	0.0448
Triglycerides in small VLDL	27005778	0.0955	0.0325
Primary biliary cirrhosis	26394269	0.0265	0.0659
Concentration of IDL particles	27005778	0.109	0.1199
Squamous cell lung cancer	27488534	0.0923	0.0674
Serum cystatin c	26831199	-0.0761	-0.0106
Child birth weight	23202124	-0.0544	-0.0059
Phospholipids in IDL	27005778	0.1337	0.1094
Mean Accumbens	25607358	-0.127	-0.0782
Mean platelet volume	22139419	-0.011	-0.0623
Serumurate overweight	25811787	0.0689	0.0849
Triglycerides in medium VLDL	27005778	0.0969	-0.0017
Free cholesterol in IDL	27005778	0.103	0.117
Mean Putamen	25607358	0.0452	0.0759
Tyrosine	27005778	0.0244	0.0894
Description of average fatty acid chain length; not actual carbon num	27005778	0.026	0.1208
Free cholesterol in large LDL	27005778	0.1108	0.121
Lung cancer	27488534	0.0696	0.0568
Mean Pallidum	25607358	-0.0012	0.0856
Phospholipids in very small VLDL	27005778	0.1151	0.0946
Total cholesterol in small VLDL	27005778	0.0815	0.0918
Total cholesterol in very large HDL	27005778	-0.1361	0.0768
Phospholipids in medium VLDL	27005778	0.0881	0.021
Forearm Bone mineral density	26367794	-0.1492	-0.0507
Type 2 Diabetes	22885922	0.0608	0.0084
Eczema	26482879	0.0596	0.0597
Apolipoprotein A-I	27005778	0.0506	0.0883
Isoleucine	27005778	0.0511	0.0849
Platelet count	22139419	-0.0282	-0.0447
Femoral Neck bone mineral density	26367794	-0.0562	-0.0409
HOMA-IR	20081858	-0.0443	-0.0381
Urate	23263486	0.0466	0.0253
Cholesterol esters in medium VLDL	27005778	0.0347	0.0638
Fathers age at death	27015805	-0.0158	-0.0186
Total Cholesterol	20686565	0.0349	-0.0124
Transferrin	25352340	0.0889	0.0693
Mono-unsaturated fatty acids	27005778	0.009	0.0879
Asthma	17611496	0.082	0.0285
Lung cancer (squamous cell)	24880342	0.0638	0.0464
Sleep duration	27494321	-0.0295	0.0212
Phospholipids in large VLDL	27005778	0.073	0.0211
Phospholipids in small VLDL	27005778	0.0786	0.0623
Cholesterol esters in large HDL	27005778	-0.0344	0.0347

Total lipids in small VLDL	27005778	0.0698	0.0613
Free cholesterol in small VLDL	27005778	0.0815	0.0641
Concentration of very small VLDL particles	27005778	0.0824	0.056
Mean diameter for VLDL particles	27005778	0.0711	-0.0102
Serum total triglycerides	27005778	0.0713	0.0367
Concentration of medium VLDL particles	27005778	0.0653	0.0039
Free cholesterol in very large HDL	27005778	-0.1071	0.0467
LDL cholesterol	20686565	0.0237	-0.0328
Fasting insulin main effect	22581228	-0.0517	-0.0266
2hr glucose adjusted for BMI	20081857	-0.0252	0.0925
Birth weight	27680694	-0.0195	-0.013
Lung cancer (all)	24880342	0.0215	0.0213
Glucose	27005778	-0.0732	-0.0598
Concentration of small VLDL particles	27005778	0.0658	0.0462
Leucine	27005778	0.0032	0.0545
Phospholipids in large HDL	27005778	0.0078	0.0411
Concentration of very large HDL particles	27005778	-0.0758	0.0268
Fasting proinsulin	20081858	0.0811	0.0727
Mean diameter for LDL particles	27005778	-0.0403	-0.0394
Lung adenocarcinoma	27488534	0.0143	0.0298
Lumbar spine bone mineral density	22504420	-0.0194	-0.018
Total lipids in very small VLDL	27005778	0.0709	0.065
Total lipids in large HDL	27005778	-0.0098	0.0365
Free cholesterol in medium VLDL	27005778	0.0641	0.0231
Concentration of large HDL particles	27005778	0.0018	0.0373
Total lipids in medium VLDL	27005778	0.0594	0.0083
Phospholipids in chylomicrons and largest VLDL particles	27005778	-0.0232	0.0109
Triglycerides in large VLDL	27005778	0.061	-0.0048
Total lipids in very large HDL	27005778	-0.0774	0.0774
Triglycerides in chylomicrons and largest VLDL particles	27005778	-0.0083	0.0232
Difference in height between childhood and adulthood; age 8	23449627	0.0564	-0.0022
Acetate	27005778	0.0298	0.0481
Free cholesterol in large HDL	27005778	-0.0116	0.046
Celiac disease	20190752	0.0053	0.0554
Fasting glucose main effect	22581228	-0.0038	-0.009
Inflammatory Bowel Disease (Euro)	26192919	0.0322	0.0279
Phenylalanine	27005778	0.046	0.0421
Phospholipids in very large HDL	27005778	-0.0442	0.0535
Omega-9 and saturated fatty acids	27005778	-0.0104	0.0699
Mean Thalamus	25607358	0.0155	0.0123
Parents age at death	27015805	-0.0169	0.0083
Total cholesterol in medium VLDL	27005778	0.0424	0.0425
Crohns disease	26192919	0.0244	-0.0011
Glycoprotein acetyls; mainly a1-acid glycoprotein	27005778	0.0123	0.0169
Heart rate	23583979	-0.0143	-0.0274

Total lipids in large VLDL	27005778	0.0456	-3.00E-04
HOMA-B	20081858	0.0227	0.0381
Concentration of large VLDL particles	27005778	0.044	0.0036
Concentration of chylomicrons and largest VLDL particles	27005778	-0.0017	0.0018
Femoral neck bone mineral density	22504420	-0.0014	0.017
Concentration of very large VLDL particles	27005778	-0.013	-0.0126
Total cholesterol in large VLDL	27005778	0.005	8.00E-04
Free cholesterol in large VLDL	27005778	0.0187	0.0062
Triglycerides in very large VLDL	27005778	-0.0085	-0.0111
Total lipids in chylomicrons and largest VLDL particles	27005778	-0.037	0.0069
Alzheimers disease	24162737	-0.0055	-0.0164
Cholesterol esters in large VLDL	27005778	0.0264	0.0188
Phospholipids in very large VLDL	27005778	-2.27E-05	0.022
Total lipids in very large VLDL	27005778	-0.0243	0.0092
Ischemic stroke	26935894	NA	NA

rg.3393	rg.4803_11	p.2247_1	p.2257	p.3393	p.4803_11	minp
0.7584	0.5816	0	0	6.99E-34	4.85E-25	0
0.3719	0.4385	0	0	5.95E-10	2.25E-14	0
1	0.5365	6.99E-34	5.95E-10	0	7.10E-08	0
0.5365	1	4.85E-25	2.25E-14	7.10E-08	0	0
0.0237	0.1034	1.98E-09	8.70E-35	0.6999	0.1118	8.70E-35
0.0815	0.1542	4.38E-12	2.00E-23	0.2881	0.0415	2.00E-23
0.0173	-0.2105	4.42E-08	1.17E-19	0.8057	0.0088	1.17E-19
-0.1199	0.1255	7.39E-08	5.11E-15	0.1539	0.0794	5.11E-15
-0.1475	0.0254	0.0182	1.83E-12	0.0031	0.6221	1.83E-12
0.0316	0.0326	0.0046	2.25E-09	0.7847	0.7956	2.25E-09
-0.0697	0.0922	0.017	1.96E-08	0.4184	0.3151	1.96E-08
0.0671	0.163	2.07E-07	1.06E-07	0.4023	0.0364	1.06E-07
-0.2542	-0.1136	1.65E-07	7.08E-07	2.67E-05	0.0447	1.65E-07
0.0179	0.1669	9.65E-07	1.67E-07	0.8088	0.0185	1.67E-07
-0.1007	0.184	6.00E-04	2.56E-05	0.3127	0.0502	2.56E-05
0.0979	0.0245	3.09E-05	0.0344	0.0693	0.62	3.09E-05
-0.0249	0.2082	0.005	3.15E-05	0.784	0.0148	3.15E-05
0.1472	0.0446	3.83E-05	0.0635	0.0043	0.4117	3.83E-05
0.0777	0.0386	9.70E-05	0.0587	0.143	0.4315	9.70E-05
0.2157	-0.0178	2.00E-04	0.0762	0.0024	0.8006	2.00E-04
-0.1013	0.0675	0.0315	3.00E-04	0.2365	0.3506	3.00E-04
-0.1408	-0.2238	0.0153	0.531	0.0625	4.00E-04	4.00E-04
0.0212	0.0099	5.00E-04	7.00E-04	0.7424	0.8806	5.00E-04
0.2212	0.1049	5.00E-04	0.0031	0.0017	0.1267	5.00E-04
0.0952	0.0104	5.00E-04	0.0513	0.0735	0.8502	5.00E-04
-0.0019	-0.1789	8.00E-04	0.0037	0.9914	0.3093	8.00E-04
-0.207	-0.1461	9.00E-04	0.4538	0.007	0.0229	9.00E-04
0.092	0.3671	0.001	0.0144	0.463	0.002	0.001
-0.0534	0.3017	0.0063	0.0012	0.7112	0.036	0.0012
0.1205	0.1492	0.0016	0.5427	0.2361	0.1516	0.0016
0.1881	0.0495	0.0017	0.1762	0.019	0.5208	0.0017
-0.0533	0.1953	0.9962	0.5455	0.3702	0.0018	0.0018
0.117	0.03	0.0018	0.2827	0.0633	0.6776	0.0018
-0.1889	-0.0891	0.1088	0.7055	0.0019	0.1295	0.0019
-0.2205	-0.1397	0.0019	0.7891	0.0019	0.0235	0.0019
-0.0657	-0.3309	0.2061	0.8468	0.6027	0.0026	0.0026
-0.3628	-0.049	0.1215	0.4095	0.0027	0.6711	0.0027
-0.0218	-0.3242	0.406	0.6595	0.8558	0.0027	0.0027
0.1309	0.0311	0.0039	0.056	0.0063	0.5342	0.0039
0.2281	0.1505	0.3146	0.6983	0.0039	0.0557	0.0039
0.0665	0.0063	0.004	0.1187	0.2294	0.9065	0.004
0.2251	0.001	0.0044	0.0116	0.1809	0.9944	0.0044
-0.1603	0.2143	0.7796	0.334	0.0434	0.0061	0.0061
-0.0667	0.2216	0.7955	0.2106	0.3765	0.0068	0.0068

0.2184	0.0839	0.0078	0.1347	0.0312	0.3798	0.0078
-0.1016	-0.105	0.1599	0.5683	0.0203	0.0092	0.0092
0.1367	0.0856	0.0101	0.0695	0.423	0.6088	0.0101
0.1335	0.0841	0.0101	0.069	0.432	0.6157	0.0101
0.1333	0.1497	0.1268	0.4167	0.0259	0.0132	0.0132
-0.0119	-0.2956	0.7469	0.3135	0.9293	0.0138	0.0138
-0.1906	0.0489	0.0142	0.4989	0.0482	0.6782	0.0142
-0.0437	0.151	0.8491	0.2302	0.4543	0.0142	0.0142
0.0419	0.2889	0.6121	0.5455	0.7605	0.0175	0.0175
-0.2407	-0.0072	0.0188	0.756	0.0409	0.9434	0.0188
-0.076	-0.0254	0.0193	0.3745	0.6096	0.8479	0.0193
0.4961	-0.1992	0.1336	0.7661	0.0194	0.2158	0.0194
0.0982	0.003	0.0207	0.7866	0.1964	0.9666	0.0207
0.129	0.0411	0.0229	0.482	0.1037	0.6345	0.0229
-0.0396	0.2922	0.7308	0.5966	0.7338	0.0234	0.0234
0.0405	0.1487	0.0234	0.3192	0.7042	0.1968	0.0234
0.2439	-0.2939	0.165	0.1529	0.0455	0.0243	0.0243
-0.105	-0.1528	0.1844	0.1675	0.1177	0.0255	0.0255
-0.1344	-0.1517	0.0277	0.5513	0.1098	0.0447	0.0277
-0.0706	-0.0379	0.1466	0.0314	0.2018	0.4566	0.0314
-0.0965	-0.0061	0.3287	0.0332	0.1631	0.9266	0.0332
-0.1782	-0.1609	0.04	0.5254	0.2831	0.3005	0.04
0.1061	-0.1045	0.1948	0.0409	0.2171	0.2485	0.0409
-0.0932	-0.0643	0.076	0.3372	0.0444	0.2328	0.0444
-0.1928	0.0221	0.2354	0.4497	0.0457	0.8162	0.0457
-0.1492	-0.2766	0.0998	0.8165	0.2582	0.0478	0.0478
0.2412	0.0132	0.2665	0.9714	0.0491	0.9104	0.0491
0.1776	-0.0067	0.1099	0.0493	0.2248	0.964	0.0493
0.1413	-0.0167	0.1433	0.0514	0.3189	0.9032	0.0514
0.2043	0.0351	0.0917	0.0527	0.186	0.8238	0.0527
0.2401	-0.2684	0.2442	0.1602	0.0744	0.0527	0.0527
0.0502	-0.1085	0.4346	0.0543	0.5424	0.2636	0.0543
0.1853	0.0388	0.2646	0.055	0.3639	0.8307	0.055
-0.0442	-0.1946	0.8806	0.9501	0.6796	0.0554	0.0554
0.0479	0.2261	0.2088	0.4232	0.6951	0.0587	0.0587
-0.0952	-0.2318	0.1947	0.5846	0.455	0.0611	0.0611
0.1645	-0.0112	0.1412	0.0623	0.2708	0.9379	0.0623
-0.1892	0.0238	0.1484	0.0664	0.1116	0.8464	0.0664
-0.0058	-0.3146	0.4645	0.9081	0.9736	0.0683	0.0683
-0.1627	-0.0229	0.2454	0.0724	0.216	0.8561	0.0724
-0.0431	0.1084	0.7462	0.2302	0.4662	0.074	0.074
0.0234	0.1724	0.2516	0.0772	0.89	0.3254	0.0772
-0.005	0.1368	0.3055	0.2372	0.9524	0.0796	0.0796
-0.0916	0.026	0.4208	0.0803	0.5278	0.8472	0.0803
0.0455	-0.1137	0.7036	0.4075	0.411	0.0818	0.0818

-0.0188	-0.1057	0.0836	0.6244	0.8506	0.2586	0.0836
0.1845	0.0449	0.1248	0.0839	0.2306	0.7773	0.0839
0.1171	0.2384	0.1383	0.2475	0.3682	0.0842	0.0842
0.2145	0.1288	0.0864	0.7126	0.1484	0.3925	0.0864
0.185	0.009	0.1577	0.0866	0.2313	0.9527	0.0866
0.1986	0.0168	0.8253	0.0871	0.1526	0.9048	0.0871
0.1657	-0.0041	0.171	0.0901	0.27	0.9781	0.0901
-0.0097	0.0596	0.0903	0.1527	0.936	0.6258	0.0903
0.1832	4.00E-04	0.1673	0.0906	0.2161	0.9978	0.0906
-0.0705	0.0991	0.2362	0.6223	0.2614	0.0916	0.0916
-0.0359	0.1686	0.0924	0.5062	0.7835	0.2316	0.0924
0.1869	-0.0187	0.1744	0.096	0.2102	0.9025	0.096
0.0616	-0.3156	0.7991	0.9546	0.7406	0.0965	0.0965
0.0386	-0.2427	0.2909	0.8338	0.8067	0.0985	0.0985
0.1911	0.0943	0.2451	0.7316	0.0986	0.4611	0.0986
-0.1086	0.2227	0.897	0.9128	0.4673	0.0997	0.0997
-0.2137	-0.0668	0.4447	0.6413	0.1049	0.5945	0.1049
0.0584	-0.262	0.2948	0.966	0.7254	0.1108	0.1108
0.1709	-0.0374	0.2362	0.1112	0.2558	0.8117	0.1112
0.0586	-0.2729	0.3887	0.9896	0.7347	0.1117	0.1117
0.185	-0.0699	0.2757	0.1154	0.2462	0.6452	0.1154
0.1425	-0.001	0.1966	0.1175	0.3409	0.9951	0.1175
0.0076	-0.2333	0.1821	0.8731	0.9609	0.1188	0.1188
0.1534	-0.0053	0.2163	0.1189	0.3082	0.9727	0.1189
0.0208	-0.1599	0.2614	0.122	0.8975	0.3681	0.122
0.0385	0.0405	0.1263	0.2657	0.7298	0.7178	0.1263
0.0855	0.0443	0.6975	0.1267	0.6718	0.8188	0.1267
-0.0362	0.0592	0.2382	0.1285	0.6776	0.4531	0.1285
-0.0796	-0.2043	0.8625	0.4113	0.5475	0.1287	0.1287
0.1308	-0.1678	0.3661	0.498	0.1986	0.1329	0.1329
0.1832	0.0012	0.2029	0.1361	0.2391	0.9938	0.1361
0.0272	-0.0182	0.1365	0.4014	0.6239	0.7506	0.1365
-0.1216	-0.0602	0.3127	0.185	0.1365	0.4805	0.1365
-0.1694	-0.1575	0.4644	0.4407	0.1466	0.1573	0.1466
-0.1835	-0.1713	0.7624	0.4682	0.1489	0.1762	0.1489
0.0418	-0.0926	0.3296	0.2885	0.4341	0.1503	0.1503
-0.0137	-0.0848	0.4497	0.4218	0.8169	0.1534	0.1534
0.009	-0.0415	0.1542	0.3305	0.9249	0.6704	0.1542
0.1409	0.0275	0.4833	0.1559	0.3223	0.8509	0.1559
-0.0512	-0.2013	0.5414	0.8409	0.7006	0.157	0.157
0.0756	-0.0596	0.4061	0.1582	0.6151	0.7041	0.1582
-0.2337	-0.0119	0.6588	0.6558	0.161	0.9346	0.161
0.0349	-0.2162	0.8992	0.8484	0.8203	0.1639	0.1639
-0.0152	0.0761	0.4566	0.1687	0.9202	0.5712	0.1687
0.0628	-0.0278	0.3112	0.1704	0.6707	0.855	0.1704

0.0235	-0.1472	0.2463	0.4832	0.8441	0.1711	0.1711
0.1557	0.0187	0.1931	0.1712	0.3213	0.9059	0.1712
0.0546	0.0386	0.1719	0.3269	0.5757	0.6859	0.1719
0.0981	0.0623	0.1723	0.5807	0.4198	0.6088	0.1723
0.0068	-0.1043	0.6597	0.1793	0.9456	0.2922	0.1793
0.0605	-7.00E-04	0.2618	0.18	0.6785	0.9965	0.18
0.1845	0.0354	0.1839	0.3561	0.1963	0.7949	0.1839
0.0268	0.0738	0.184	0.819	0.7669	0.3895	0.184
-0.0759	0.1348	0.3772	0.9162	0.4453	0.1841	0.1841
0.1415	0.0929	0.1856	0.2413	0.3544	0.5464	0.1856
0.0984	-0.1969	0.1869	0.3809	0.5392	0.2808	0.1869
-0.0696	-0.017	0.8251	0.1911	0.4118	0.8342	0.1911
0.0395	0.0374	0.3584	0.1913	0.7137	0.6386	0.1913
0.0821	0.0494	0.1918	0.9785	0.5161	0.7165	0.1918
0.1071	0.0692	0.2733	0.1936	0.4496	0.6236	0.1936
-0.09	-0.0431	0.4567	0.1964	0.3331	0.6667	0.1964
-0.0463	-0.1712	0.7853	0.2443	0.7405	0.1974	0.1974
0.0498	-0.1207	0.7887	0.1996	0.752	0.4057	0.1996
0.1487	0.0347	0.2758	0.2027	0.3328	0.8276	0.2027
-0.0765	-0.0254	0.2067	0.2434	0.3911	0.7886	0.2067
-0.176	-0.0483	0.9878	0.2481	0.208	0.7081	0.208
0.0241	-0.0038	0.2085	0.2521	0.8584	0.9783	0.2085
0.0951	0.0049	0.3392	0.2093	0.4825	0.9723	0.2093
-0.3034	-0.2506	0.2794	0.4919	0.2105	0.2325	0.2105
0.0815	0.0591	0.2124	0.7211	0.5007	0.6389	0.2124
0.0852	-0.0644	0.2158	0.6312	0.6742	0.7182	0.2158
0.0324	-0.0687	0.2176	0.8485	0.6705	0.4412	0.2176
-0.1015	0.1483	0.3787	0.3339	0.4077	0.2181	0.2181
-0.0392	-0.1813	0.52	0.2262	0.7971	0.2279	0.2262
0.0761	-0.0431	0.5355	0.2332	0.5966	0.7609	0.2332
0.0823	0.0773	0.515	0.2539	0.2355	0.2418	0.2355
-0.0327	-0.0177	0.2395	0.3937	0.6917	0.8219	0.2395
-0.1539	0.0187	0.543	0.5873	0.2401	0.876	0.2401
0.0389	0.0425	0.2445	0.4218	0.5022	0.4288	0.2445
-0.0164	-0.0303	0.6036	0.2467	0.8856	0.7899	0.2467
0.1191	0.0278	0.8084	0.7786	0.2493	0.7969	0.2493
0.072	-0.0207	0.3519	0.7313	0.2494	0.7522	0.2494
0.0697	-0.0058	0.2519	0.3979	0.532	0.9588	0.2519
0.0346	0.1526	0.9179	0.2755	0.8157	0.2579	0.2579
0.1263	0.0816	0.2712	0.6481	0.3596	0.5571	0.2712
0.1684	0.0785	0.4514	0.5793	0.2766	0.632	0.2766
-0.034	-0.0822	0.5174	0.6146	0.682	0.2798	0.2798
-0.0035	0.0075	0.2826	0.7085	0.9763	0.9491	0.2826
0.0722	0.0168	0.2845	0.3111	0.5527	0.892	0.2845
-0.1263	-0.1317	0.6158	0.5311	0.3121	0.2851	0.2851

0.0627	9.46E-05	0.3163	0.2881	0.593	0.9994	0.2881
0.1237	0.0346	0.2889	0.3156	0.3147	0.7819	0.2889
0.0077	-0.0217	0.2907	0.4099	0.9465	0.8569	0.2907
0.0571	0.0757	0.2908	0.8587	0.6193	0.5173	0.2908
0.0856	0.0623	0.2928	0.5231	0.4688	0.5824	0.2928
0.0332	0.0094	0.3004	0.9433	0.7666	0.9302	0.3004
-0.1723	-0.1377	0.3051	0.579	0.3603	0.4208	0.3051
0.0712	-0.0738	0.5741	0.4073	0.3109	0.3093	0.3093
-0.1068	0.0213	0.3404	0.6217	0.3095	0.8265	0.3095
-0.1422	0.0935	0.792	0.3127	0.3889	0.5595	0.3127
0.0305	0.0543	0.5472	0.6692	0.618	0.3141	0.3141
-0.1013	0.1109	0.7284	0.7038	0.3171	0.323	0.3171
-0.0232	-0.0909	0.3203	0.3983	0.861	0.4298	0.3203
0.0729	0.0092	0.3319	0.4099	0.5288	0.936	0.3319
0.047	-0.1892	0.9772	0.5512	0.7887	0.3347	0.3347
-0.0795	-0.1144	0.9088	0.4637	0.5228	0.3432	0.3432
-0.1706	-0.0524	0.4128	0.7242	0.3448	0.7364	0.3448
0.1053	0.1066	0.3453	0.4907	0.4101	0.4737	0.3453
-0.1924	-0.0212	0.7164	0.7021	0.3552	0.9092	0.3552
-0.1207	-0.1387	0.863	0.6987	0.4249	0.3569	0.3569
-0.0692	-0.0212	0.6251	0.6568	0.3588	0.7583	0.3588
-0.0048	-0.0362	0.3932	0.3651	0.9688	0.7734	0.3651
-0.0964	-0.1083	0.8832	0.506	0.43	0.3658	0.3658
0.0911	0.0544	0.3667	0.6988	0.4553	0.6653	0.3667
-0.0893	-0.1073	0.9787	0.4932	0.4612	0.3675	0.3675
0.0448	-0.0084	0.3684	0.8769	0.698	0.9425	0.3684
0.1108	-0.0276	0.7485	0.8665	0.3689	0.8156	0.3689
0.0304	0.0205	0.3693	0.9335	0.7928	0.8627	0.3693
-0.1378	-0.0728	0.4261	0.3705	0.4543	0.6565	0.3705
0.1169	0.0361	0.9123	0.7395	0.3719	0.7769	0.3719
-0.0532	-0.023	0.3725	0.969	0.604	0.8301	0.3725
0.1271	0.1411	0.7731	0.5829	0.4245	0.3733	0.3733
-0.1018	-0.1123	0.8729	0.4251	0.4156	0.3796	0.3796
0.0426	0.0838	0.9411	0.3874	0.7204	0.3873	0.3873
-0.076	-0.0076	0.9385	0.85	0.3875	0.9228	0.3875
-0.0397	0.0528	0.3893	0.4286	0.561	0.3962	0.3893
0.1583	0.0547	0.6594	0.6429	0.3972	0.7394	0.3972
-0.0979	-0.0402	0.5836	0.4225	0.4999	0.7713	0.4225
-0.0013	0.0499	0.912	0.4231	0.9936	0.7234	0.4231
0.1066	0.0853	0.8608	0.8753	0.4301	0.5275	0.4301
-0.1062	0.0898	0.811	0.903	0.4656	0.4357	0.4357
0.0301	0.0229	0.5349	0.4519	0.7978	0.8467	0.4519
-0.0542	-0.0185	0.5186	0.9773	0.4547	0.7669	0.4547
0.0128	0.1021	0.8809	0.8199	0.9262	0.4593	0.4593
-0.0179	0.0542	0.7341	0.4599	0.8048	0.4742	0.4599

0.0113	-0.0289	0.4787	0.9957	0.9189	0.795	0.4787
-0.0599	0.0661	0.7166	0.4835	0.5968	0.5196	0.4835
0.0223	-0.0113	0.4971	0.9498	0.8436	0.9184	0.4971
0.0619	-0.0785	0.9815	0.9799	0.6226	0.5135	0.5135
-0.0385	-0.0166	0.9702	0.6458	0.5431	0.8137	0.5431
0.0526	-0.0701	0.8523	0.8449	0.6737	0.5508	0.5508
0.0395	0.0677	0.9388	0.9889	0.7359	0.5622	0.5622
0.0325	0.0639	0.7646	0.9105	0.7752	0.5646	0.5646
0.0665	0.0502	0.8929	0.8411	0.566	0.6462	0.566
0.0662	-0.003	0.6009	0.9112	0.5853	0.9783	0.5853
0.0318	0.0654	0.9423	0.823	0.8113	0.6019	0.6019
0.0272	0.0541	0.681	0.7247	0.8072	0.6074	0.6074
0.0431	0.0483	0.9997	0.7123	0.72	0.6798	0.6798
0.0293	-0.0113	0.6953	0.8656	0.7994	0.917	0.6953
NA	NA	NA	NA	NA	NA	NA

padj
0
0
0
0
8.28E-32
1.90E-20
1.12E-16
4.85E-12
1.73E-09
2.13E-06
1.86E-05
9.98E-05
0.00015576
0.000157754
0.024158532
0.0290769
0.02958086
0.0359637
0.090986
0.1874
0.2808
0.374
0.467
0.467
0.467
0.7448
0.837
0.929
1
1
1
1
1
1
1
1
1
1
1
1
1
1
1
1

1
1
1
1
1
1
1
1
1
1
1
1
1
1
NA
