# Supplementary material for: Biological insights from multi-omic analysis of 31 genomic risk loci for adult hearing difficulty
Source: PLoS Genet. 2020 Sep 28;16(9):e1009025. doi: 10.1371/journal.pgen.1009025 (PMC7544108; doi:10.1371/journal.pgen.1009025)
Supplement: Attachment — Submitted filename: Response to Reviewers 2020-07-03.docx [file pgen.1009025.s034.docx]

**Kalra et al. Response to Reviewers**

**Editor’s Comments**

“In addition to the comments provided by the reviewers, we ask that you include some discussion regarding the validity of "hearing aid usage".  It is well known that most (estimates are as high as 80%) people who could benefit from hearing aids will not wear them, even if they have them.  This seems to be more of an attitude issue, which could still be under genetic, albeit different genes, control.”

We appreciate the editor’s comment that hearing aid use reflects both hearing difficulty and a behavioral choice by the individual and we have added both new results and discussion sections to more explicitly consider this. Overall, our analyses indicate a substantial shared genetic component between hearing difficulty and hearing aid use. This is supported by a new analysis showing that heritable risk for hearing aid use is enriched in genomic regions of open chromatin in cochlear epithelial cells, similar to hearing difficulty (p. 9, paragraph 2; Table S20). That said, the genetic correlation between hearing difficulty and hearing aid use is well below 1, and it seems likely that the behavioral choice has a distinct genetic etiology. Indeed, a glimmer of this effect can be detected in our genetic correlation analyses. We found a positive genetic correlation between increased risk for hearing difficulty and increased risk for major depression disorder (rg = 0.20, p = 6.0e-4), which may reflect that ARHI is a well-known risk factor for depression in older adults. However, there was no significant genetic correlation between risk for hearing aid use and risk for depression, and intriguingly the trend is toward a negative genetic correlation (rg = -0.10, p > 0.05). Though speculative, it is possible that the subset of hearing-impaired individuals who use hearing aids are genetically predisposed toward resilience or that hearing aid use ameliorates these adverse impacts. We have added a paragraph in the Discussion explaining all of these points.

**Reviewer #1**

“The authors have put together a comprehensive manuscript describing their GWAS using the UKBB questionnaire-based information on hearing. To my knowledge there exists no prospective data set of individuals appropriately screened and tested for ARHI that would provide the power necessary for significance. These authors, utilizing this database, have displayed a powerful tool for GWAS and have identified and replicated several genomic regions associated with ARHI. Furthermore, these authors demonstrate the homology to our best genetic model thus far, the mouse. I think this is a meaningful paper that will serve hearing research well.”

We thank the reviewer for his positive feedback!

**Reviewer #2:**

“The work is generally carried out at a high standard but I am a bit conflicted how far the study advances the field conceptually. GWAS studies (at least one using the same data set) have already identified many loci associated with hearing difficulties in the adult and are replicated here. The current analysis adds genes to the list without substantially changing the overall picture. The contribution of each locus to phenotype is small, which is in agreement with earlier findings. Perhaps one of the most interesting parts of the paper is the attempt to link omics studies in mice with mapping data in humans to begin a functional characterization of the putative risk loci. Based on these data, the authors conclude that many of the genes linked to hearing loss are apparently expressed in the inner ear and in sensory structures. This is also in agreement with previous studies indicating that genetic forms of hearing loss frequently affect genes expressed in sensory structures of the inner ear. While the datasets are certainly of value for the community and worthwhile reporting, functional validation is largely missing. This is further confounded by limitations of the study. Hearing phenotypes were self-reported without quantitative assessment. Most of the identified loci have not been replicated in independent cohorts. The authors also point out that the data are a ‘best guess’ and need additional validation. Thus, while the data are certainly interesting, I find they fall short of the kind of advance expected for PLOS Genetics.”

We thank the reviewer for their positive comments regarding the quality of the work. With respect to novelty and impact, we believe it is helpful to compare our paper to systems genetics studies of other traits. In the field of psychiatric genetics, many people would have hypothesized that risk for schizophrenia is primarily enriched in gene regulatory regions for cortical neurons. However, when the landmark GWAS of schizophrenia was published, revealing 108 risk loci (Ripke et al. 2014, *Nature*), it was difficult to test this hypothesis, due to a lack of multi-omic data in the brain. Generating these multi-omic data and using them to model the specific gene regulatory perturbations underlying risk for schizophrenia became perhaps the central thrust of research in the field. Each new paper provided a piece of the puzzle, a specific dataset or analysis that pushed the field forward. Notably, many of these seminal systems genetics papers were published in top journals such as *Nature* (e.g., Won et al. 2016)*, Science* (Li et al. 2018)*,* and *Cell* (de la Torre-Ubieta, et al. 2018). Each of these papers has been viewed as a seminal advance, since it helps to define the etiology and mechanisms, despite the fact that many of the causal genes and mechanisms remain elusive. We believe our paper -- the first systems genetics analysis of hearing difficulty GWAS -- makes a similarly important contribution within the field of adult hearing loss. In revising the Discussion, we have sought to clarify both the potential impact and limitations of our data and analyses and to chart a path forward.
